# Low-Density Lipoprotein Cholesterol, Cardiovascular Disease Risk, and Mortality in China

**DOI:** 10.1001/jamanetworkopen.2024.22558

**Published:** 2024-07-18

**Authors:** Liang Chen, Shi Chen, Xueke Bai, Mingming Su, Linkang He, Guangyu Li, Guangda He, Yang Yang, Xiaoyan Zhang, Jianlan Cui, Wei Xu, Lijuan Song, Hao Yang, Wenyan He, Yan Zhang, Xi Li, Shengshou Hu

**Affiliations:** 1 National Clinical Research Center for Cardiovascular Diseases, State Key Laboratory of Cardiovascular Disease, Fuwai Hospital, National Center for Cardiovascular Diseases, Chinese Academy of Medical Sciences and Peking Union Medical College, Beijing, China; 2 Department of Cardiac Surgery, Fuwai Hospital, National Center for Cardiovascular Diseases, Chinese Academy of Medical Sciences and Peking Union Medical College, Beijing, China; 3 Fuwai Hospital Chinese Academy of Medical Sciences, Shenzhen, Shenzhen, China; 4 Central China Sub-center of the National Center for Cardiovascular Diseases, Zhengzhou, China

## Abstract

**Importance:**

Limited evidence supports the association between low-density lipoprotein cholesterol (LDL-C) and mortality across different atherosclerotic cardiovascular disease (ASCVD) risk stratifications.

**Objective:**

To explore the associations between LDL-C levels and mortality and to identify the optimal ranges of LDL-C with the lowest risk of mortality in populations with diverse ASCVD risk profiles.

**Design, Setting, and Participants:**

The ChinaHEART project is a prospective cohort study that recruited residents aged 35 to 75 years from 31 provinces in mainland China between November 2014 and December 2022. Participants were categorized into low-risk, primary prevention, and secondary prevention cohorts on the basis of their medical history and ASCVD risk. Data analysis was performed from December 2022 to October 2023.

**Main Outcomes and Measures:**

The primary end point was all-cause mortality, and secondary end points included cause-specific mortality. Mortality data were collected from the National Mortality Surveillance System and Vital Registration. The association between LDL-C levels and mortality was assessed by using Cox proportional hazard regression models with various adjusted variables.

**Results:**

A total of 4 379 252 individuals were recruited, and 3 789 025 (2 271 699 women [60.0%]; mean [SD] age, 56.1 [10.0] years) were included in the current study. The median (IQR) LDL-C concentration was 93.1 (70.9-117.3) mg/dL overall at baseline. During a median (IQR) follow-up of 4.6 (3.1-5.8) years, 92 888 deaths were recorded, including 38 627 cardiovascular deaths. The association between LDL-C concentration and all-cause or cardiovascular disease (CVD) mortality was U-shaped in both the low-risk cohort (2 838 354 participants) and the primary prevention cohort (829 567 participants), whereas it was J-shaped in the secondary prevention cohort (121 104 participants). The LDL-C levels corresponding to the lowest CVD mortality were 117.8 mg/dL in the low-risk group, 106.0 mg/dL in the primary prevention cohort, and 55.8 mg/dL in the secondary prevention cohort. The LDL-C concentration associated with the lowest all-cause mortality (90.9 mg/dL vs 117.0 mg/dL) and CVD mortality (87 mg/dL vs 114.6 mg/dL) were both lower in individuals with diabetes than in individuals without diabetes in the overall cohort.

**Conclusions and Relevance:**

This study found that the association between LDL-C and mortality varied among different ASCVD risk cohorts, suggesting that stricter lipid control targets may be needed for individuals with higher ASCVD risk and those with diabetes.

## Key Points

**Question:**

What is the association between low-density lipoprotein cholesterol (LDL-C) and mortality in patients with different atherosclerotic cardiovascular disease (ASCVD) risk stratifications?

**Findings:**

In this cohort study involving 3 789 025 participants, a U-shaped association was detected in both the low-risk and primary prevention cohorts, and a J-shaped association was detected in the secondary prevention cohort.

**Meaning:**

These findings suggest that lipid management strategies should be differentially performed in individuals at different ASCVD risk levels.

## Teaser

This cohort study examines the associations between low-density lipoprotein cholesterol levels and mortality among populations with diverse atherosclerotic cardiovascular disease (CVD) risks in China.

## Introduction

Low-density lipoprotein cholesterol (LDL-C) is considered one of the leading risk factors for cardiovascular disorders, primarily owing to its causal relationship with atherosclerotic cardiovascular disease (ASCVD).^1,2^ High LDL-C levels are estimated to cause up to 4.3 million deaths annually, accounting for 7.7% of deaths worldwide.^3^ Thus, LDL-C has been robustly proven to be a treatment target in randomized clinical trials. Lipid-lowering treatments, especially statins, have been widely applied as basic clinical strategies for primary and secondary prevention of ASCVD.^4^

The U-shaped association between LDL-C concentration and all-cause or cardiovascular disease (CVD) mortality has recently been well defined in the general population of developed countries.^5,6,7^ Nonetheless, this association in individuals from developing countries and in populations with different ASCVD risk stratifications remains unclear. The different associations between LDL-C and mortality in individuals with different ASCVD risks could determine the different lipid management strategies that are used in clinical practice, which is relevant for billions of people. The primordial prevention of CVD has been promoted worldwide, and lipid management is one of the important aspects of this strategy. However, the association between LDL-C and mortality in the low-ASCVD-risk population in clinical practice is unclear.^8^ In addition, the strategy of the lower, the better for lipid-lowering treatment in patients with ASCVD requires more support because the evidence from specialized ASCVD trials is limited owing to the small sample size,^9,10^ and the evidence from a large cohort in general population is needed. Furthermore, study on the management of dyslipidemia along with comorbidities including diabetes and hypertension remains insufficient.^11,12^

In this study, we leveraged a nationwide, population-based, prospective cohort from the China Health Evaluation and Risk Reduction through Nationwide Teamwork (ChinaHEART) project, with an aim to compare LDL-C profiles in China with those in other Western countries and to determine the association between LDL-C concentration and mortality in populations with different ASCVD risks. In addition, we further explored this association in different subgroups according to age, sex, and comorbidity status, especially diabetes.

## Methods

### Study Design and Population

The study cohort was based on the ChinaHEART project, which is an ongoing government-funded public health project in China. Details of the project design have been previously described.^13^ In brief, from November 2014 to December 2022, 353 study sites (including districts in urban areas or counties in rural areas) scattered throughout all 31 provinces of mainland China were selected to represent the diversity in geographic distribution, demographic structure, health behavior profiles, metabolic characteristics, and disease patterns. At each study site, residents aged 35 to 75 years who had lived in the community for at least 6 of the prior 12 months were invited and recruited.

Among the 4 379 252 participants enrolled in ChinaHEART project, 524 906 individuals (12.0%) with nonfasting blood lipid measurements and 65 321 (1.5%) with missing LDL-C measurements were excluded from the current study. The included participants were divided into 3 cohorts by ASCVD risk status: (1) participants with established ASCVD (such as a history of ischemic stroke, myocardial infarction, peripheral arterial disease, coronary artery bypass grafting, or percutaneous coronary intervention), which was termed the secondary prevention cohort; (2) participants with high CVD risk (an estimated 10-year risk for ASCVD of more than 10% according to the China-PAR risk algorithm^14^) but no established ASCVD, which was termed the primary prevention cohort; and (3) the other cohorts, termed the low-risk cohort (eFigure 1 in Supplement 1).

The ChinaHEART project was approved by the central ethics committee of Fuwai Hospital. All of the enrolled participants provided written informed consent. The study followed the Strengthening the Reporting of Observational Studies in Epidemiology (STROBE) reporting guidelines.

### Data Collection and Variables

For each participant, standardized in-person interviews were conducted by trained personnel using an electronic data acquisition system with a real-time logical check function. More specifically, participants’ sociodemographic characteristics (including sex, age, occupation, education, household income, marital status, and medical insurance status), health behaviors (including smoking and alcohol consumption), and medical history (including self-reported hypertension and diabetes) were collected (see more in the eAppendix in Supplement 1). Participants underwent laboratory tests and physical examinations under uniform equipment and standardized procedures. The details are described in the eAppendix in Supplement 1.

### End Points

The primary end point of this study was all-cause mortality during the follow-up. Participants’ vital status and causes of death were collected through a passive follow-up process up to December 2022, in which a linkage of data was established between the cohort and the National Mortality Surveillance System and Vital Registration of the China Center for Disease Control and Prevention, which covers urban and rural areas in all 31 provinces of mainland China.^13,15^ The death records in this system are reported by health care institutions nearly in real time and are subsequently checked against local residential and health insurance records on an annual basis.

In the National Mortality Surveillance System and Vital Registration, the main cause of death was coded by using the *International Statistical Classification of Diseases and Related Health Problems, Tenth Revision (ICD-10)*. CVD (*ICD-10* codes I00-I90) and cancer (*ICD-10 *codes C00-C96), as well as specific types, including ischemic heart disease, cerebrovascular disease, and lung cancer, were included as study outcomes in our analyses. A detailed list of end points with *ICD-10* codes is available in the eAppendix in Supplement 1.

### Statistical Analysis

Data analysis was performed from December 2022 to October 2023. We used the frequency and percentage for categorical variables and the mean (SD) or median (IQR) for continuous variables. The Kaplan-Meier curves represented all causes, CVD, and cancer death among the 3 cohorts. We predefined 7 categories of LDL-C (<40, 40-70, 70-100, 100-130, 130-160, 160-190, and >190 mg/dL; to convert LDL-C to nanomoles per liter, multiply by 0.0259), with 100 to 130 mg/dL as the reference group. The association between LDL-C levels and mortality was assessed by using Cox proportional hazard regression models with various adjusted variables: model 1 included age and sex, model 2 included education level and annual household income, and model 3 included current smoking status, alcohol status, systolic blood pressure, high-density lipoprotein cholesterol, triglyceride, lipid-lowering treatment, and medical history, including diabetes, chronic obstructive pulmonary disease (COPD), and cancer. No violation of the proportional hazard assumptions was observed in the scaled Schoenfeld residuals graphs. For cause-specific mortality, the cause-specific hazard model was used to analyze the competing risks.

Furthermore, restricted cubic splines (RCSs) based on the Cox model with 4 predefined variables (5th, 35th, 65th, and 95th percentiles) were calculated to estimate the nonlinear association between LDL-C and patient outcomes. Afterward, 2-line segmented linear models were fitted to quantify the associations. Initially, we used the estimated values from the RCS model and LDL-C values to construct a regression model. Subsequently, we examined the presence of change points by exploring all of the potential locations and selecting the location with the highest likelihood.^16^ Moreover, the hazard ratios (HRs) and 95% CIs per 1 mmol/L increase in both directions of the change point were estimated by using the Cox model.

Stratified analysis was conducted to evaluate the heterogeneities in associations between LDL-C and mortality across subgroups by ASCVD risk group, as well as age (<60 years vs ≥60 years), sex, and medical history of diabetes or hypertension. We added interaction terms to investigate the effect modification of age, sex, diabetes, and hypertension. The *P* values of the interaction terms were evaluated via the false discovery rate.

Further details of the statistical analyses are provided in the Appendix in Supplement 1. A 2-sided *P* < .05 was considered to indicate statistical significance. All of the analyses used SAS statistical software version 9.4 (SAS Institute) and R statistical software version 4.1.2 (R Project for Statistical Computing), with the packages survminer, rms, segmented, and SmoothHR.

## Results

### Baseline Characteristics of Participants

This study enrolled a total of 3 789 025 participants (2 271 699 women [60.0%]; mean [SD] age, 56.1 [10.0] years), including 2 838 354 individuals in the low-risk cohort, 829 567 in the primary prevention cohort, and 121 104 in the secondary prevention cohort of patients with ASCVD. The baseline characteristics of the participants are presented in the Table and eTables 1, 2, and 3 in Supplement 1. The majority of the participants resided in rural areas (2 284 120 participants [60.3%]), 705 797 (18.6%) had an annual household income over 50 000 yuan (as of June 17, 2024, 1 yuan = US $0.14), and 294 640 (7.8%) had a college education or higher. Metabolic disorders, including diabetes and obesity, were the most common comorbidities. The proportions of patients with malignant diseases, such as cancer, COPD, and chronic kidney disease (CKD), were low in all of the groups.

**Table.** Baseline Characteristics of ChinaHEART Population in Different Risk Stratification Groups

Characteristic	Participants, No. (%)			
	Low-risk population (n = 2 838 354)	Primary prevention (n = 829 567)	Secondary prevention (n = 121 104)	Total (N = 3 789 025)
Age, mean (SD), y	53.3 (9.2)	64.6 (6.9)	61.9 (8.1)	56.1 (10.0)
Sex				
Male	987 521 (34.8)	468 170 (56.4)	61 635 (50.9)	1 517 326 (40.0)
Female	1 850 833 (65.2)	361 397 (43.6)	59 469 (49.1)	2 271 699 (60.0)
Urbanity				
Urban	1 148 514 (40.5)	307 450 (37.1)	48 941 (40.4)	1 504 905 (39.7)
Rural	1 689 840 (59.5)	522 117 (62.9)	72 163 (59.6)	2 284 120 (60.3)
Region				
South	1 863 525 (65.7)	343 481 (41.4)	56 857 (46.9)	2 263 863 (59.7)
North	974 829 (34.3)	486 086 (58.6)	64 247 (53.1)	1 525 162 (40.3)
Education				
Primary school	1 204 544 (42.4)	436 477 (52.6)	57 488 (47.5)	1 698 509 (44.8)
Middle school	931 998 (32.8)	245 511 (29.6)	36 857 (30.4)	1 214 366 (32.0)
High school	417 834 (14.7)	104 324 (12.6)	18 174 (15.0)	540 332 (14.3)
College or above	251 405 (8.9)	35 751 (4.3)	7484 (6.2)	294 640 (7.8)
Unknown	32 573 (1.1)	7504 (0.9)	1101 (0.9)	41 178 (1.1)
Annual household income, yuan^a^				
<10 000	442 477 (15.6)	185 147 (22.3)	24 939 (20.6)	652 563 (17.2)
10 000-50 000	1 562 175 (55.0)	451 658 (54.4)	66 371 (54.8)	2 080 204 (54.9)
>50 000	566 167 (19.9)	119 118 (14.4)	20 512 (16.9)	705 797 (18.6)
Unknown	267 535 (9.4)	73 644 (8.9)	9282 (7.7)	350 461 (9.2)
Marital status				
Married	2 664 429 (93.9)	748 616 (90.2)	110 267 (91.1)	3 523 312 (93.0)
Unmarried	143 980 (5.1)	73 090 (8.8)	9805 (8.1)	226 875 (6.0)
Unknown	29 945 (1.1)	7861 (0.9)	1032 (0.9)	38 838 (1.0)
Health insurance status				
Insured	2 771 612 (97.6)	812 644 (98.0)	118 768 (98.1)	3 703 024 (97.7)
Uninsured	10 607 (0.4)	1860 (0.2)	183 (0.2)	12 650 (0.3)
Unknown	56 135 (2.0)	15 063 (1.8)	2153 (1.8)	73 351 (1.9)
Life behavior				
Current smoker	480 785 (16.9)	235 606 (28.4)	27 063 (22.3)	743 454 (19.6)
Current drinker	631 943 (22.3)	230 627 (27.8)	28 330 (23.4)	890 900 (23.5)
Lipids, median (IQR), mg/dL				
Low-density lipoprotein cholesterol	91.9 (70-115.8)	98.2 (75.7-122.9)	87.3 (62.9-114.3)	93.1 (70.9-117.3)
High-density lipoprotein	54.5 (44.9-66.5)	49.1 (41-59.2)	49.5 (41.4-60.3)	53 (43.7-65)
Total cholesterol	171.7 (146.6-199.5)	177.1 (150.4-206.1)	165.5 (136.5-197.2)	172.9 (146.9-200.7)
Triglyceride	115.1(84.1-164.7)	135.5 (95.7-195.7)	126.7 (92.1-182.5)	119.6 (86.8-171.8)
Blood pressure, median (IQR), mm Hg				
Systolic	129.0 (119.0-140.0)	151.5 (139.0-165.0)	143.0 (130.0-158.5)	133.5 (121.5-148.0)
Diastolic	79.5 (72.5-86.0)	86.0 (78.5-94.0)	83.0 (75.5-91.0)	80.5 (73.5-88.0)
Body mass index, median (IQR)^b^	24.2(22.1-26.4)	25.6 (23.4-28)	25.5 (23.3-27.7)	24.5 (22.4-26.9)
Waist circumference, median (IQR), cm	82 (76-89)	88 (82-95)	87 (80-94)	84 (77.5-90)
Medical history				
Diabetes	98 262 (3.5)	157 964 (19.0)	22 170 (18.3)	278 396 (7.3)
Obesity	388 379 (13.7)	208 984 (25.2)	27 789 (22.9)	625 152 (16.5)
Cancer	10 164 (0.4)	4034 (0.5)	773 (0.6)	14 971 (0.4)
Chronic obstructive pulmonary disease	5028 (0.2)	3389 (0.4)	851 (0.7)	9268 (0.2)
Chronic kidney disease	2248 (0.1)	2099 (0.3)	1119 (0.9)	5466 (0.1)
Lipid-lowering treatment	47 512 (1.7)	41 110 (5.0)	24 852 (20.5)	113 474 (3.0)

SI conversion factors: To convert high-density lipoprotein to nanomoles per liter, multiply by 0.0259; low-density lipoprotein cholesterol to nanomoles per liter, multiply by 0.0259; total cholesterol to nanomoles per liter, multiply by 0.0259; triglyceride to millimoles per liter, multiply by 0.0113.

^a^ As of June 17, 2024, 1 yuan = US $0.14.

^b^ Body mass index is calculated as weight in kilograms divided by height in meters squared.

The median (IQR) LDL-C concentration was 93.1 (70.9-117.3) mg/dL overall at baseline. The median LDL-C level was greater in women than in men, although there was no obvious change trend across the different age groups. The use of lipid-lowering treatment was 1.7% (47 512 participants) in the low-risk cohort, 5.0% (41 110 participants) in the primary prevention cohort, and 20.5% (24 852 participants) in the secondary prevention cohorts; however, the proportions of participants who achieved the LDL-C control target according to the latest guidelines were 85.2% (2 417 683 participants) in the low-risk cohort, 52.0% (431 504 participants) in the primary prevention cohort, and 31.9% (38 584 participants) in the secondary prevention cohorts. The distribution of LDL-C according to different risk stratifications is shown in eFigure 2 in Supplement 1. In addition, the distribution of LDL-C concentrations was similar in individuals with or without malignant diseases at baseline (eFigure 2 in Supplement 1).

### LDL-C and Mortality in the Overall Population

During a median (IQR) follow-up of 4.6 (3.1-5.8) years, 92 888 participants (2.45%) died, including 44 977 (1.58%) in the low-risk cohort, 41 217 (4.97%) in the primary prevention cohort, and 6694 (5.53%) in the secondary prevention cohort. The causes of death varied among the 3 cohorts, with CVD (38 627 deaths) and cancer being the most common causes (eTable 4 in Supplement 1). The CVD mortality rates were 0.54% (15 331 participants) in the low-risk cohort, 2.33% (19 341 participants) in the primary prevention cohort, and 3.27% (3955 participants) in the secondary prevention cohort.

**Figure 1.**
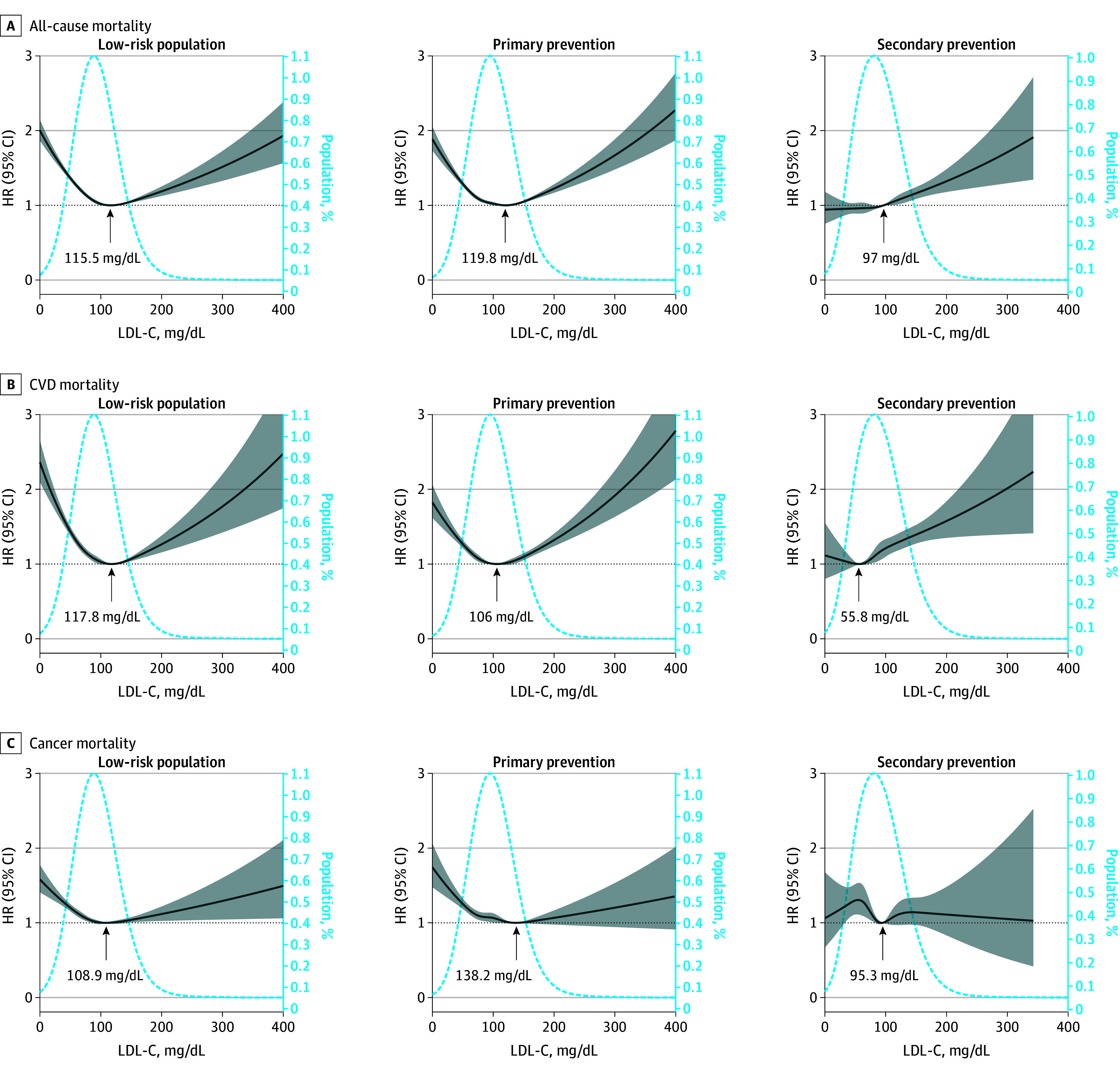
Associations Between Low-Density Lipoprotein Cholesterol (LDL-C) and All-Cause, Cardiovascular Disease (CVD), and Cancer Mortality Graphs show multivariate adjusted hazard ratios (HRs; solid lines) and 95% CIs (shaded areas) derived from restricted cubic spline regressions with 4 knots for all-cause (A), CVD-related (B), and cancer-related (C) mortality in different atherosclerotic cardiovascular disease risk groups according to LDL-C levels on a continuous scale. Dashed lines show the fraction of the population with different LDL-C levels. Arrows indicate the concentration of LDL-C with the lowest risk of mortality. Analyses used the variables in model 3. To convert LDL-C to nanomoles per liter, multiply by 0.0259.

The mortality rates and risks of individuals with different LDL-C levels among different cohorts are presented in eTable 5 in Supplement 1. The association between LDL-C levels and the risk of all-cause CVD mortality was U-shaped in both the low-risk and primary prevention cohorts but J-shaped in the secondary prevention cohort (Figure 1). For the secondary prevention cohort, for all-cause mortality, the 95% CI included an HR of 1.0 at LDL-C concentrations lower than 70 mg/dL, which increased significantly with increasing LDL-C (eFigure 3 in Supplement 1). The trend in the association between LDL-C and CVD mortality was consistent with that for all-cause mortality, as described above.

**Figure 2.**
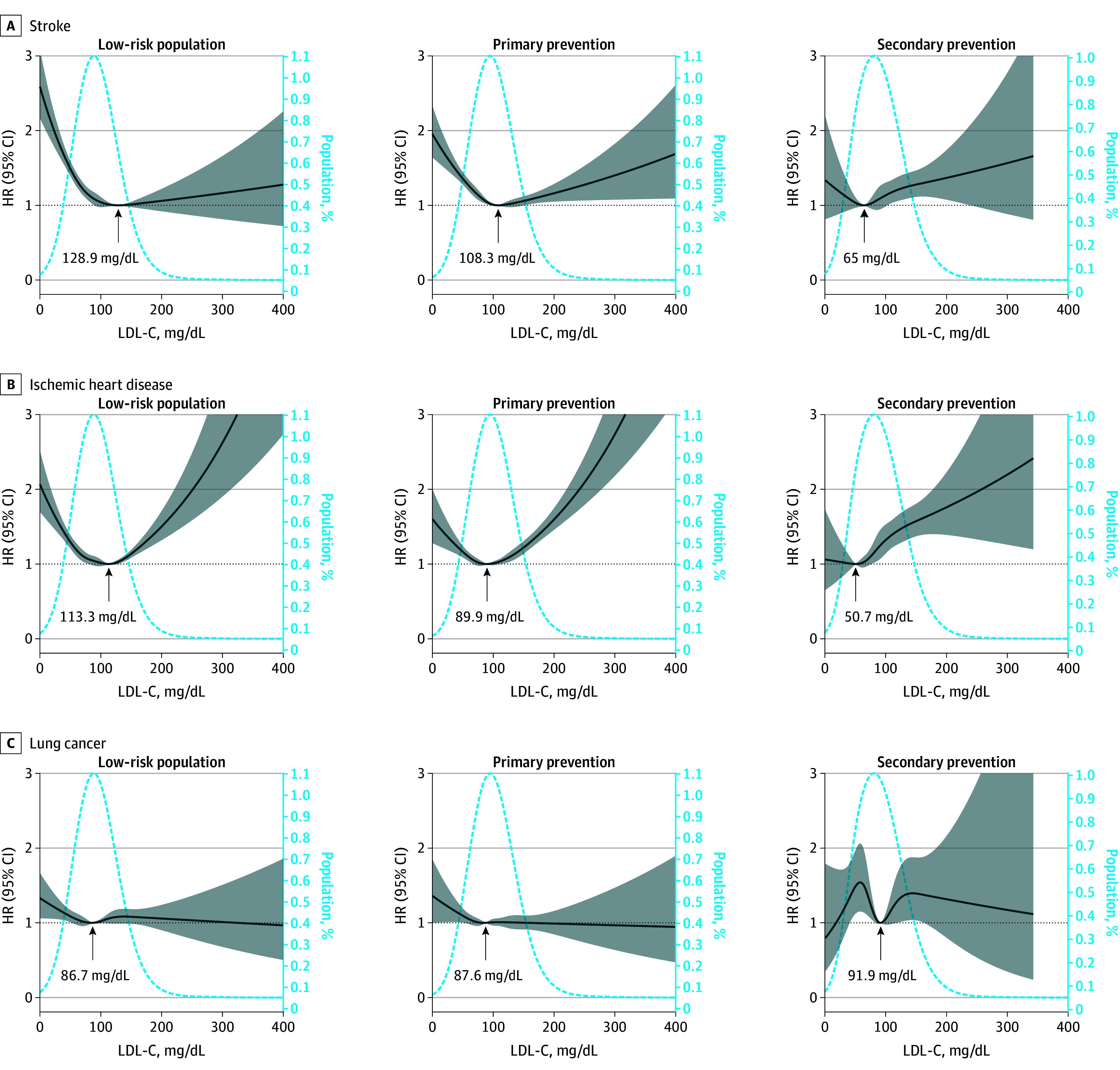
Associations Between Low-Density Lipoprotein Cholesterol (LDL-C) and Cause-Specific Mortality Graphs show multivariate adjusted hazard ratios (HRs; solid lines) and 95% CIs (shaded areas) derived from restricted cubic spline regressions with 4 knots for mortality related to stroke (A), ischemic heart disease (B), and lung cancer (C) in different atherosclerotic cardiovascular disease risk groups according to LDL-C levels on a continuous scale. Dashed lines show the fraction of the population with different LDL-C levels. Arrows indicate the concentration of LDL-C with the lowest risk of mortality. Analyses used the variables in model 3. To convert LDL-C to nanomoles per liter, multiply by 0.0259.

Regarding the association between LDL-C and cause-specific mortality, a U-shaped association was found between LDL-C and stroke and between LDL-C and ischemic heart disease in the low-risk and primary prevention cohorts; moreover, a monotonic increasing association was present in the secondary prevention cohort. The lowest RCS curves for stroke (128.9 mg/dL vs 108.3 mg/dL vs 65 mg/dL) and ischemic heart disease mortality (113.3 mg/dL vs 89.9 mg/dL vs 50.7 mg/dL) were lower in the higher ASCVD risk cohorts vs the lower risk cohorts (Figure 2). For lung cancer, the 95% CI included an HR of 1.0 at any concentration of LDL-C.

### LDL-C and Mortality According to Stratification

The LDL-C levels corresponding to the lowest CVD mortality from the RCS were 117.8 mg/dL in the low-risk cohort, 106.0 mg/dL in the primary prevention cohort, and 55.8 mg/dL in the secondary prevention cohort, which indicates that lower LDL-C targets with increasing ASCVD risk should be considered for reducing CVD mortality (Figure 1). The trends in the association between LDL-C and all-cause mortality in the secondary prevention cohort were different between male and female individuals (eFigure 4 in Supplement 1). Similarly, in the secondary prevention cohort, high LDL-C levels were associated with increased all-cause or CVD mortality risk in male individuals, whereas this association was not detected in women.

In the low-risk cohort, LDL-C was associated with all-cause mortality, and CVD mortality differed between individuals who were younger and older than 60 years (eFigure 5 in Supplement 1). In the lowest LDL-C group (LDL-C <40 mg/dL), elderly individuals had a greater HR for all-cause mortality (HR, 1.68; 95% CI, 1.59-1.78) than middle-aged individuals (HR, 1.41; 95% CI, 1.32-1.52). Nonetheless, for the highest LDL-C group (LDL-C >190 mg/dL), the HR of all-cause mortality for elderly individuals was lower (HR, 1.07; 95% CI, 0.96-1.20) than that for middle-aged individuals (HR, 1.65; 95% CI, 1.46-1.87).

**Figure 3.**
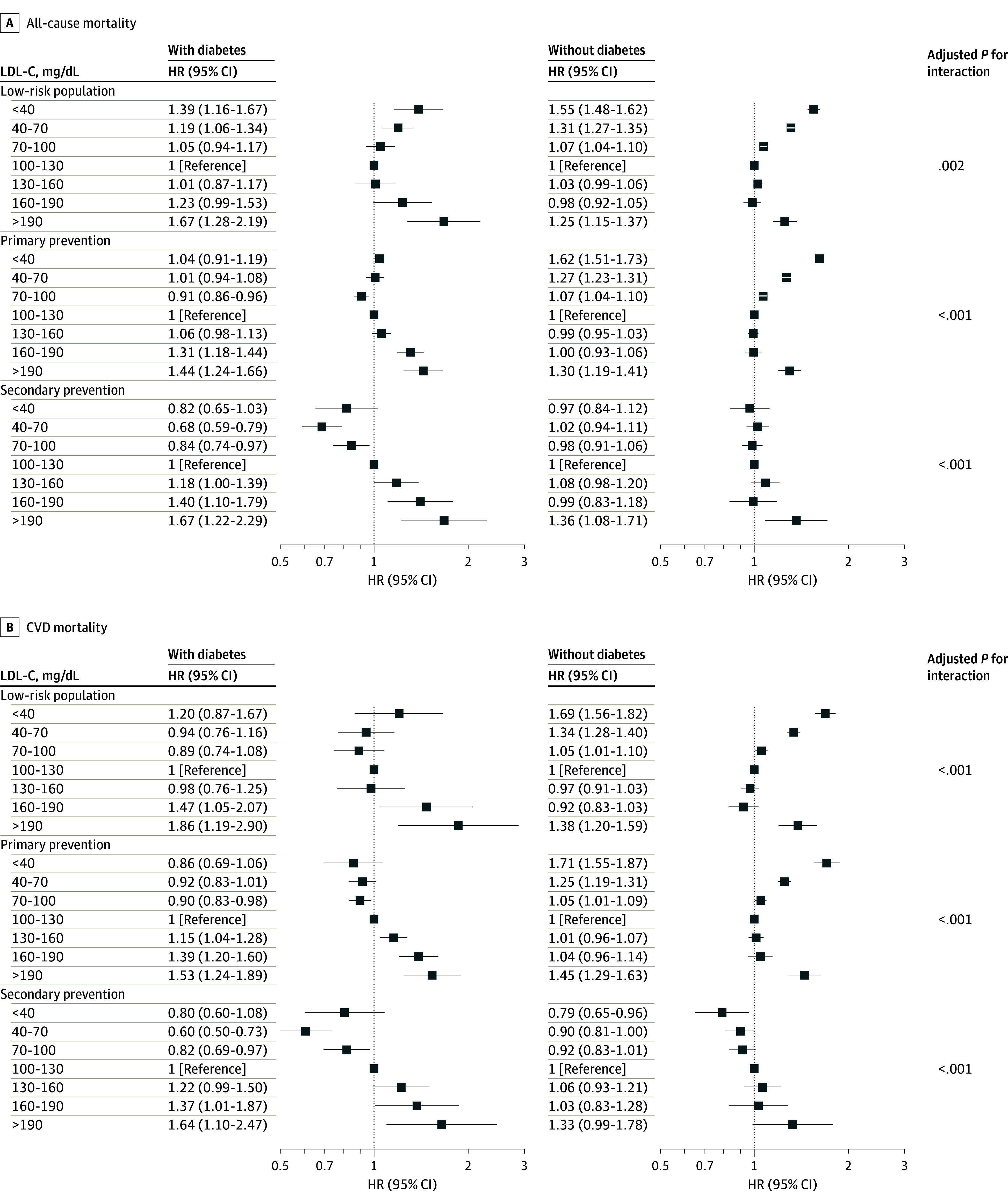
Associations Between Low-Density Lipoprotein Cholesterol (LDL-C) and All-Cause and Cardiovascular Disease (CVD) Mortality in Different Atherosclerotic Cardiovascular Disease Risk Groups Stratified by Diabetes Status The multivariable adjusted analyses used the variables in model 3 except diabetes. HR indicates hazard ratio. To convert LDL-C to nanomoles per liter, multiply by 0.0259.

In participants with or without hypertension, the association between LDL-C and mortality was consistent with that in the overall population (eFigure 6 in Supplement 1). In the overall cohort, the LDL-C concentration associated with the lowest all-cause mortality (90.9 mg/dL vs 117.0 mg/dL) and CVD mortality (87.0 mg/dL vs 114.6 mg/dL) were both lower in individuals with diabetes than in individuals without diabetes (eFigure 7 in Supplement 1). The interaction effects between LDL-C levels and diabetes status on all-cause mortality and CVD mortality were significant in each ASCVD risk cohort (*P* for interaction <.05). Compared with individuals without diabetes, the risk of all-cause and CVD mortality was greater in individuals with diabetes with LDL-C concentrations greater than 160 mg/dL in all ASCVD risk stratifications. These findings suggested that diabetes is a nonnegligible risk factor for all individuals, regardless of their ASCVD risk (Figure 3).

### Sensitivity Analyses

**Figure 4.**
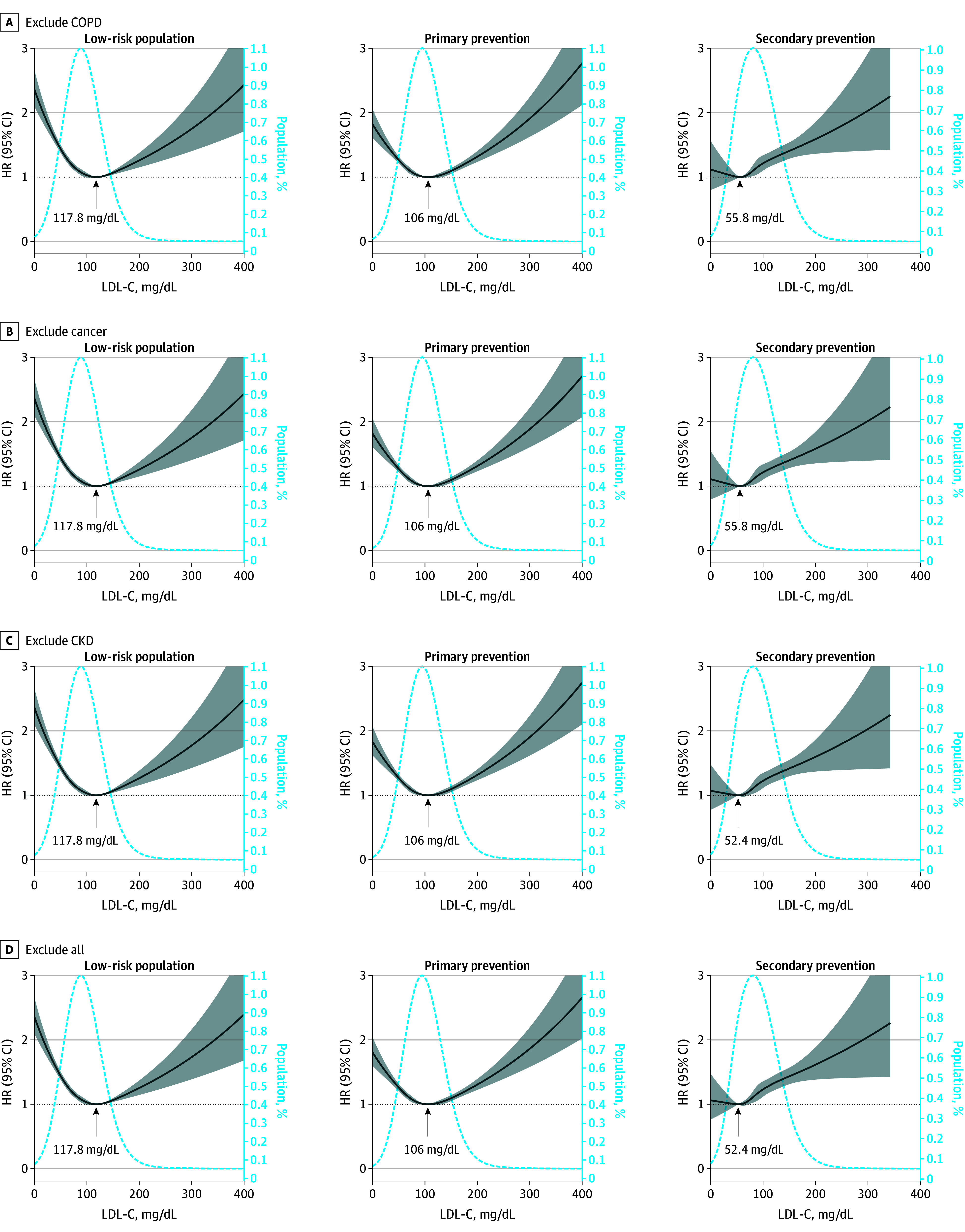
Associations Between Low-Density Lipoprotein Cholesterol (LDL-C) and Cardiovascular Disease Mortality in Different Atherosclerotic Cardiovascular Disease Risk Groups With Exclusion of Individuals With Baseline Chronic Disease Graphs show multivariate adjusted hazard ratios (HRs; solid lines) and 95% CIs (shaded areas). Dashed lines show the fraction of the population with different LDL-C levels. Arrows indicate the concentration of LDL-C with the lowest risk of mortality. Analyses used the variables in model 3. To convert LDL-C to nanomoles per liter, multiply by 0.0259. CKD indicates chronic kidney disease; COPD, chronic obstructive pulmonary disease.

We applied nonlinear splines and change point detection to estimate the associations and risk turning points between mortality and LDL-C on a continuous scale. These results also validated the U-shaped association (eFigure 8 and eTable 6 in Supplement 1). Dyslipidemia and mortality can both be affected by comorbidities and cachexia. To minimize reverse causality, such as the body mass index paradox, we excluded individuals with chronic diseases such as COPD, CKD, and cancer at enrollment, and the associations between LDL-C and CVD or all-cause mortality were consistent with the main results (Figure 4 and eFigure 9 in Supplement 1).

We assessed the effects of time and residual confounders on this association via sensitivity analyses. An analysis excluding the first 3 years of follow-up was also performed (eFigure 10 in Supplement 1), and the results after excluding these populations were also similar to those of the main analyses. To further evaluate the potential drug treatment effect, we excluded individuals who received lipid-lowering treatment at baseline or adjusted their LDL-C concentration based on standard equations. The association between LDL-C level and mortality did not change in this sensitivity analysis (eFigures 11 and 12 in Supplement 1).

## Discussion

### Principal Findings

To our knowledge, this cohort study is the first to evaluate the association of LDL-C with mortality across different ASCVD risk populations based on a study of 4.4 million individuals from a nationwide, general population–based, prospective cohort in China. The general LDL-C level was lower in our overall Chinese cohort than in Western countries; however, there was a greater proportion of total CVD mortality in China than in Western countries.^17^ U-shaped associations between LDL-C levels and the risk of all-cause or CVD mortality in low-risk and primary prevention cohorts and a J-shaped association were observed in the secondary prevention population. The LDL-C level with the lowest risk of all-cause or CVD mortality was lower in the population with higher ASCVD risk and individuals with diabetes.

### Possible Explanation of the Association

For the first time, our study divided the study population by 10-year ASCVD risk and ASCVD status instead of the general population and demonstrated distinct associations between LDL-C levels and mortality. The positive association between LDL-C concentration and mortality has been widely recognized in previous studies^5,18,19,20,21,22^ and was the same in our low-risk and primary prevention cohorts. We found that the LDL-C concentration associated with the lowest risk of all-cause mortality in these populations in China was much lower than that in Western countries.^5^ Regarding the association between extremely low LDL-C levels and all-cause or CVD mortality, we observed the opposite association, as in previous studies.^5,6,7,18^ In addition, our results suggested that this inverse association in individuals with low LDL-C was attenuated as the ASCVD risk increased and disappeared in the secondary prevention cohort.

On the one hand, the levels of LDL-C and stroke-related mortality, especially hemorrhagic stroke (HS), had an inverse association (L-shaped). Stroke incidence and mortality are much greater in China than in Western countries,^23^ and HS accounts for an equal number of deaths as ischemic stroke in China.^17,24^ Multiple levels of evidence have demonstrated the association between low levels of LDL-C and increased risk of HS.^19,25,26,27,28,29,30,31^ On the other hand, the association between low levels of LDL-C and the increased risk of mortality could be explained by reverse causation of severe disease burden, especially during a relatively short follow-up period. Some diseases or debilitation that have been hypothesized to result in low cholesterol are associated with poor prognosis.^32^ We tried to confirmed that the inverse association at a low LDL-C level did not present a significant decrease when individuals with COPD, CKD, or cancer were excluded.^33^ However, the reverse causality related to frailty and sarcopenia cannot be ruled out because they are also recognized as risk factors for dyslipidemia.

### Populations With Different CVD Risks

The concept of preventing the development of ASCVD risk factors is known as primordial prevention, which has been recently recommended by the American Heart Association.^34^ However, most lipid-lowering treatments in clinical trials have been conducted for the primary and secondary prevention of ASCVD,^5,35^ and clinical evidence for the use of statins in low-risk populations is lacking. Our study demonstrated that elevated levels of LDL-C above the change points (approximately 110 mg/dL) were associated with increased risks of CVD mortality and all-cause mortality in the low-risk population, which suggested the urgency for intensive lipid management as early in life as possible, as recommended by Braunwald.^36^

In the secondary prevention population, individuals with LDL-C above 70 mg/dL had increased risk of all-cause and CVD mortality, which is highly consistent with the guidelines,^37^ whereas those with LDL-C less than 70 mg/dL did not show significantly increased or decreased risk. The clinical benefits of extremely low levels of LDL-C on CVD or mortality have remained controversial.^38,39,40,41^ Our findings suggest that extremely low levels of LDL-C seem safe for the secondary prevention population in China, although the incremental efficacy remains to be verified in further clinical trials.

### Clinical Implications for Lipid Management

In China, only a very small fraction of people who could benefit from LDL-C reduction receive lipid-lowering medications.^42,43^ Because CVD mortality is the leading cause of mortality in China, lipid-lowering treatments to reduce ASCVD events will greatly increase life expectancy. For the secondary prevention population, consistent with the recommendations of guidelines,^37^ patients with LDL-C less than 70 mg/dL had the lowest risk of all-cause and CVD mortality. More importantly, our study demonstrated that an extremely low LDL-C level was not associated with increased mortality, thus indicating the safety of intensive lipid-lowering therapy for patients with preexisting ASCVD. In addition, individuals with diabetes should receive more intensive lipid monitoring and reduction.^44^ The net benefit of extremely low LDL-C remains to be elucidated, especially for the Chinese population with a high proportion of HS-related mortality. Given the inevitable effects of reverse causality, the reverse association at a lower LDL-C could not be used as an argument against lipid reduction in ASCVD prevention.

More importantly, our study emphasized the importance of lipid management for primordial prevention, in which individuals with a low risk of ASCVD but a high LDL-C are also recommended to receive lipid-lowering medications or health behavior education. Taken together, these findings indicate that the association between LDL-C levels and mortality differs across populations with stratified ASCVD risks; however, higher LDL-C levels are associated with increased all-cause mortality and CVD mortality among these populations.

### Limitations

This study had several limitations. First, our datasets lacked indicators of frailty and sarcopenia, which could affect both LDL-C concentration and mortality. Thus, their specific effects on the association between LDL-C and mortality should be further excluded in the future. Second, despite the application of various sensitivity analysis methods to mitigate the potential impact of underlying health conditions and severe morbid states under baseline conditions, the potential for reverse causality remains unavoidable. Third, the LDL-C levels that were included in this study were measured at baseline, thereby precluding the exclusion of subsequent influences from health behavior interventions, pharmacotherapeutic regimens, and other pertinent factors that may engender bias in the observed association between LDL-C levels and mortality outcomes.

## Conclusions

In this study, the associations between LDL-C concentrations and mortality differed across populations with a stratified risk of ASCVD. In low-risk and primary prevention cohorts, both low and high levels of LDL-C were associated with increased CVD, and the LDL-C level associated with the lowest mortality in these populations was lower than that in Western populations. For the secondary prevention cohort, extremely low LDL-C levels were not associated with increased mortality. This study demonstrated the urgent need for lipid-lowering treatment for individuals with high LDL-C levels not only in traditional primary and secondary prevention settings but also in populations at low risk of ASCVD. Individuals with diabetes in low-risk, primary and secondary prevention populations should receive more intensive lipid monitoring and reduction.

## Notes

Supplement 1. **eAppendix.** Supplementary methods **eTable 1.** Baseline characteristics of low-risk population **eTable 2.** Baseline characteristics of primary prevention population **eTable 3.** Baseline characteristics of secondary prevention population **eTable 4.** Distribution of cause of death, overall and by different ASCVD risk groups **eTable 5.** Associations of LDL-C levels with all cause, CVD, and cancer mortality in different ASCVD risk groups (mg/dL) **eTable 6.** Estimated change points in the association between LDL-C and mortality in different ASCVD risk groups, and associations with mortality below and above the change point, from piecewise two-line models **eFigure 1.** The flowchart of study design **eFigure 2.** Baseline LDL-C distribution in different subgroups **eFigure 3.** Hazard ratio for all cause (A) and CVD (B) mortality according to categories of LDL-C with different covariates adjusted **eFigure 4.** Association between LDL-C and all cause (A), CVD (B) mortality in different ASCVD risk groups by sex **eFigure 5.** Association between LDL-C and all cause (A), CVD (B) mortality in different ASCVD risk groups by age **eFigure 6.** Association between LDL-C and all cause (A), CVD (B) mortality in different ASCVD risk groups by hypertension **eFigure 7.** Associations between LDL-C and all-cause and CVD mortality stratified by diabetes status **eFigure 8.** Spline models of the association between LDL-C and all-cause, CVD, and cancer mortality in different ASCVD risk groups **eFigure 9.** Association between LDL-C and all cause mortality in different ASCVD risk groups exclusion of individual with baseline chronical disease **eFigure 10.** Association between LDL-C and all cause, CVD, and cancer mortality in different ASCVD risk groups after exclusion of individual with less than 3-year follow-up **eFigure 11.** Association between adjusted LDL-C and all-cause, CVD, and cancer mortality in different ASCVD risk groups **eFigure 12.** Association between LDL-C and all-cause, CVD, and cancer mortality in different ASCVD risk groups after excluding lipid-lowering treatment
eReferences

Supplement 2.
Data Sharing Statement
