# Supplementary material for: Low-Density Lipoprotein Cholesterol, Cardiovascular Disease Risk, and Mortality in China
Source: JAMA Netw Open. 2024 Jul 18;7(7):e2422558. doi: 10.1001/jamanetworkopen.2024.22558 (PMC11258592; doi:10.1001/jamanetworkopen.2024.22558)
Supplement: Supplement 1. — eAppendix. Supplementary methods eTable 1. Baseline characteristics of low-risk population eTable 2. Baseline characteristics of primary prevention population eTable 3. Baseline characteristics of secondary prevention population eTable 4. Distribution of cause of death, overall and by different ASCVD risk groups eTable 5. Associations of LDL-C levels with all cause, CVD, and cancer mortality in different ASCVD risk groups (mg/dL) eTable 6. Estimated change points in the association between LDL-C and mortality in different ASCVD risk groups, and associations with mortality below and above the change point, from piecewise two-line models eFigure 1. The flowchart of study design eFigure 2. Baseline LDL-C distribution in different subgroups eFigure 3. Hazard ratio for all cause (A) and CVD (B) mortality according to categories of LDL-C with different covariates adjusted eFigure 4. Association between LDL-C and all cause (A), CVD (B) mortality in different ASCVD risk groups by sex eFigure 5. Association between LDL-C and all cause (A), CVD (B) mortality in different ASCVD risk groups by age eFigure 6. Association between LDL-C and all cause (A), CVD (B) mortality in different ASCVD risk groups by hypertension eFigure 7. Associations between LDL-C and all-cause and CVD mortality stratified by diabetes status eFigure 8. Spline models of the association between LDL-C and all-cause, CVD, and cancer mortality in different ASCVD risk groups eFigure 9. Association between LDL-C and all cause mortality in different ASCVD risk groups exclusion of individual with baseline chronical disease eFigure 10. Association between LDL-C and all cause, CVD, and cancer mortality in different ASCVD risk groups after exclusion of individual with less than 3-year follow-up eFigure 11. Association between adjusted LDL-C and all-cause, CVD, and cancer mortality in different ASCVD risk groups eFigure 12. Association between LDL-C and all-cause, CVD, and cancer mortality in different ASCVD risk grou [file jamanetwopen-e2422558-s001.pdf]

Supplemental Online Content

Chen L, Chen S, Bai X, et al. Low -density lipoprotein cholesterol, cardiovascular disease risk, and mortality in China. *JAMA Netw Open*. 2024;7(7):e2422558
doi:10.1001/jamanetworkopen.2024.22558

eAppendix. Supplementary methods
eTable 1. Baseline characteristics of low-risk population
eTable 2. Baseline characteristics of primary prevention population
eTable 3. Baseline characteristics of secondary prevention population
eTable 4. Distribution of cause of death, overall and by different ASCVD risk groups
eTable 5. Associations of LDL-C levels with all cause, CVD, and cancer mortality in different ASCVD risk groups (mg/dL)
eTable 6. Estimated change points in the association between LDL-C and mortality in different ASCVD risk groups, and associations with mortality below and above the change point, from piecewise two-line models
eFigure 1. The flowchart of study design
eFigure 2. Baseline LDL-C distribution in different subgroups
eFigure 3. Hazard ratio for all cause (A) and CVD (B) mortality according to categories of LDL-C with different covariates adjusted
eFigure 4. Association between LDL-C and all cause (A), CVD (B) mortality in different ASCVD risk groups by sex
eFigure 5. Association between LDL-C and all cause (A), CVD (B) mortality in different ASCVD risk groups by age
eFigure 6. Association between LDL-C and all cause (A), CVD (B) mortality in different ASCVD risk groups by hypertension
eFigure 7. Associations between LDL-C and all-cause and CVD mortality stratified by diabetes status
eFigure 8. Spline models of the association between LDL-C and all-cause, CVD, and cancer mortality in different ASCVD risk groups
eFigure 9. Association between LDL-C and all cause mortality in different ASCVD risk groups exclusion of individual with baseline chronical disease
eFigure 10. Association between LDL-C and all cause, CVD, and cancer mortality in different ASCVD risk groups after exclusion of individual with less than 3-year follow-up
eFigure 11. Association between adjusted LDL-C and all-cause, CVD, and cancer mortality in different ASCVD risk groups
eFigure 12. Association between LDL-C and all-cause, CVD, and cancer mortality in different ASCVD risk groups after excluding lipid-lowering treatment
eReferences

This supplemental material has been provided by the authors to give readers additional information about their work.

© 2024 Chen L et al. *JAMA Network Open.*

eAppendix. Supplementary methods

Baseline laboratory tests and physical examination

Variable	Measure methods
Total cholesterol (TC), triglyceride (TG), and high-density lipoprotein (HDL-C)	These tests were measued using a rapid lipid analyzer (CardioChek PA Analyzer; Polymer Technology Systems, Indianapolis IN, USA)
Low-density lipoprotein cholesterol (LDL-C)	LDL-C was calculated with the Sampson equation[1] (LDL-C = TC/0.948 - HDL-C/0.971 - (TG/8.56 + TG*Non-HDL-C/2140 - TG^2/16100) in mg/dL).
Blood pressures	Participants' blood pressures were measured twice on the right upper arm using an electronic blood pressure monitor (Omron HEM-7430; Omron Corporation, Kyoto, Japan) after five minutes of rest in a seated position
Fasting blood glucose	The fasting blood glucose levels were measured rapid blood glucose analyzer (BeneCheck BK6-20M Multi-Monitoring System, Suzhou PuChun Tang Biotechnology, China)
Body mass index (BMI)	BMI was calculated as weight in kilograms divided by height in meters squared

Statistical analysis

Sensitivity analyses

In the sensitivity analyses, we: 1) replotted the RCS by only including participants with 3-year or longer follow-up or those with no history of cancer or chronic obstructive pulmonary disease (COPD), to minimize reverse causality; 2) replotted RCS by excluding participants under lipid-lowering treatment or reevaluating their LDL-C levels (i.e. Those with concurrent diagnoses of ischemic heart disease or stroke were multiplied by 1.67, while those without such diagnoses were multiplied by 1.43), to take into account effects of lipid-lowering treatment; 3) explored Spline models based on Cox models for alternative non-linear associations; 4) The U-shape relationship between LDL-C and all cause and CVD death was confirmed in non-overlapping populations: those with less than 3 years of follow-up, those with 3-5 years of follow-up, and those with more than 5 years of follow-up using Cox models.

© 2024 Chen L et al. *JAMA Network Open.*

eTable 1. Baseline characteristics of low-risk population.

	**< 40 mg/dL (n=116,991)**	**40-70 mg/dL (n=532,787)**	**70-100 mg/dL (n=977,570)**	**100-130 mg/dL (n=730,335)**	**130-160 mg/dL (n=300,866)**	**160-190 mg/dL (n=84,369)**	**>190 mg/dL (n=35,436)**
Age, y	52.6(10)	52(9.6)	52.8(9.2)	54.1(8.9)	55.2(8.6)	56(8.4)	55.5(8.6)
Sex							
Male	54612 (46.7)	258246 (43.6)	356211 (36.4)	221078 (30.3)	73748 (24.5)	16626 (19.7)	7000 (19.8)
Female	62379 (53.3)	334541 (56.4)	621359 (63.6)	509257 (69.7)	227118 (75.5)	67743 (80.3)	28436 (80.2)
Urbanity							
Urban	46384 (39.6)	233630 (39.4)	392820 (40.2)	300551 (41.2)	124827 (41.5)	34968 (41.4)	15334 (43.3)
Rural	70607 (60.4)	359157 (60.6)	584750 (59.8)	429784 (58.8)	176039 (58.5)	49401 (58.6)	20102 (56.7)
Region							
South	65360 (55.9)	353032 (59.6)	631397 (64.6)	502299 (68.8)	218505 (72.6)	64685 (76.7)	28247 (79.7)
North	51631 (44.1)	239755 (40.4)	346173 (35.4)	228036 (31.2)	82361 (27.4)	19684 (23.3)	7189 (20.3)
Education							
Primary school	51620 (44.1)	250230 (42.2)	405745 (41.5)	308859 (42.3)	132152 (43.9)	38522 (45.7)	17416 (49.1)
Middle school	37895 (32.4)	198137 (33.4)	327899 (33.5)	238405 (32.6)	93901 (31.2)	25489 (30.2)	10272 (29.0)
High school	15780 (13.5)	82737 (14.0)	143260 (14.7)	110928 (15.2)	47001 (15.6)	13146 (15.6)	4982 (14.1)
College or above	10609 (9.1)	54899 (9.3)	89136 (9.1)	63595 (8.7)	24387 (8.1)	6357 (7.5)	2422 (6.8)
Unknown	1087 (0.9)	6784 (1.1)	11530 (1.2)	8548 (1.2)	3425 (1.1)	855 (1.0)	344 (1.0)
Annual household income, yuan							
<10000	19025 (16.3)	100396 (16.9)	153400 (15.7)	108351 (14.8)	44573 (14.8)	12026 (14.3)	4706 (13.3)
10000-50000	65457 (56.0)	328378 (55.4)	542337 (55.5)	400656 (54.9)	161274 (53.6)	44457 (52.7)	19616 (55.4)

© 2024 Chen L et al. *JAMA Network Open.*

>50000	21430 (18.3)	110777 (18.7)	191486 (19.6)	150503 (20.6)	65140 (21.7)	19156 (22.7)	7675 (21.7)
Unknown	11079 (9.5)	53236 (9.0)	90347 (9.2)	70825 (9.7)	29879 (9.9)	8730 (10.3)	3439 (9.7)
Marital status							
Married	110949 (94.8)	559862 (94.4)	919985 (94.1)	683150 (93.5)	279603 (92.9)	78093 (92.6)	32787 (92.5)
Unmarried	4947 (4.2)	26665 (4.5)	47310 (4.8)	39233 (5.4)	17988 (6.0)	5492 (6.5)	2345 (6.6)
Unknown	1095 (0.9)	6260 (1.1)	10275 (1.1)	7952 (1.1)	3275 (1.1)	784 (0.9)	304 (0.9)
Health insurance status							
Insured	114519 (97.9)	579148 (97.7)	954327 (97.6)	712941 (97.6)	293467 (97.5)	82524 (97.8)	34686 (97.9)
Uninsured	328 (0.3)	2009 (0.3)	3547 (0.4)	2840 (0.4)	1340 (0.4)	417 (0.5)	126 (0.4)
Unknown	2144 (1.8)	11630 (2.0)	19696 (2.0)	14554 (2.0)	6059 (2.0)	1428 (1.7)	624 (1.8)
Life behavior							
Current smoker	25465 (21.8)	124378 (21.0)	174575 (17.9)	107464 (14.7)	36447 (12.1)	8593 (10.2)	3863 (10.9)
Current drinker	29184 (24.9)	145941 (24.6)	226707 (23.2)	152221 (20.8)	56729 (18.9)	14748 (17.5)	6413 (18.1)
Lipids							
LDL, mg/dL	31.9 (24.1, 36.6)	58.5 (51, 64.6)	85.3 (78, 92.6)	112.7 (106, 120.4)	140.9 (134.9, 148.7)	170.4 (164.5, 178.1)	209.4 (197.3, 233.9)
HDL, mg/dL	61.9 (49.9, 75.8)	52.6 (42.5, 65.7)	53.8 (44.1, 65.4)	54.5 (45.6, 65.7)	55.7 (46.8, 66.9)	58 (48.3, 69.6)	61.9 (50.3, 79.7)
TC, mg/dL	116 (102.9, 126.5)	134.2 (121.4, 147.7)	163.2 (151.2, 176.3)	193.4 (181.7, 206.1)	224.3 (212.3, 238.6)	257.5 (244.4, 271.9)	307 (285, 344.5)
TG, mg/dL	119.6 (77.9, 192.2)	101.9 (74.4, 150.6)	111.6 (81.5, 158.5)	120.5 (89.5, 167.4)	127.5 (95.7, 175.4)	134.6 (99.2, 183.3)	140.8 (99.2, 201.9)
SBP, mmHg	127.5 (118, 138)	127 (117, 138)	128.5 (119, 139.5)	130 (120, 141)	131 (121, 143)	132 (121, 144)	131.5 (120.5, 144)
DBP, mmHg	79 (72, 85)	79 (72, 85.5)	79 (72.5, 86)	79.5 (73, 86.5)	80 (73, 86.5)	80 (73, 87)	80 (73, 87)
BMI, kg/m^2	23.6 (21.5, 25.9)	23.8 (21.8, 26.1)	24.1 (22.1, 26.4)	24.4 (22.4, 26.6)	24.5 (22.5, 26.7)	24.4 (22.5, 26.7)	24.3 (22.3, 26.6)

© 2024 Chen L et al. *JAMA Network Open.*

Waist, cm	81.4 (75, 88)	82 (75, 88)	82(76, 89)	82.7 (77, 89)	83 (77, 89)	83 (77, 89)	82 (77, 88)
Medical History							
Diabetes	4320 (3.7)	19830 (3.3)	31564 (3.2)	25389 (3.5)	11734 (3.9)	3743 (4.4)	1682 (4.7)
Obesity	12632 (10.8)	72796 (12.3)	133098 (13.6)	107311 (14.7)	45245 (15.0)	12261 (14.5)	5036 (14.2)
Cancer	348 (0.3)	1947 (0.3)	3296 (0.3)	2769 (0.4)	1265 (0.4)	373 (0.4)	166 (0.5)
COPD	277 (0.2)	1287 (0.2)	1761 (0.2)	1066 (0.1)	475 (0.2)	115 (0.1)	47 (0.1)
CKD	88 (0.1)	376 (0.1)	670 (0.1)	515 (0.1)	273 (0.1)	216 (0.3)	110 (0.3)
Lipid-lowering treatment	3738 (3.2)	13186 (2.2)	13825 (1.4)	9204 (1.3)	4709 (1.6)	1805 (2.1)	1045 (2.9)

LDL=Low-density lipoprotein cholesterol. HDL= High-density lipoprotein. TC=Total cholesterol. TG=Triglyceride.

SBP=Systolic blood pressure. DBP= Diastolic blood pressure. BMI=Body mass index. COPD=chronic obstructive pulmonary disease.

© 2024 Chen L et al. *JAMA Network Open.*

eTable 2. Baseline characteristics of primary prevention population.

	**< 40 mg/dL (n=21,678)**	**40-70 mg/dL (n=141,619)**	**70-100 mg/dL (n=268,207)**	**100-130 mg/dL (n=235,743)**	**130-160 mg/dL (n=112,221)**	**160-190 mg/dL (n=34,943)**	**>190 mg/dL (n=15,156)**
Age, y	64.8(7.3)	64.7(7.1)	64.6(7)	64.6(6.8)	64.5(6.8)	64.4(6.9)	63.8(7.3)
Sex							
Male	12432 (57.3)	91358 (64.5)	160706 (59.9)	127425 (54.1)	53902 (48.0)	15098 (43.2)	7249 (47.8)
Female	9246 (42.7)	50261 (35.5)	107501 (40.1)	108318 (45.9)	58319 (52.0)	19845 (56.8)	7907 (52.2)
Urbanity							
Urban	8099 (37.4)	49575 (35.0)	96027 (35.8)	88591 (37.6)	44474 (39.6)	14373 (41.1)	6311 (41.6)
Rural	13579 (62.6)	92044 (65.0)	172180 (64.2)	147152 (62.4)	67747 (60.4)	20570 (58.9)	8845 (58.4)
Region							
South	6433 (29.7)	48749 (34.4)	107741 (40.2)	103441 (43.9)	51985 (46.3)	17113 (49.0)	8019 (52.9)
North	15245 (70.3)	92870 (65.6)	160466 (59.8)	132302 (56.1)	60236 (53.7)	17830 (51.0)	7137 (47.1)
Education							
Primary school	11586 (53.4)	75586 (53.4)	141626 (52.8)	123007 (52.2)	58250 (51.9)	18336 (52.5)	8086 (53.4)
Middle school	6341 (29.3)	41805 (29.5)	79846 (29.8)	70297 (29.8)	33055 (29.5)	9987 (28.6)	4180 (27.6)
High school	2587 (11.9)	17143 (12.1)	33178 (12.4)	29914 (12.7)	14717 (13.1)	4781 (13.7)	2004 (13.2)
College or above	1038 (4.8)	6064 (4.3)	11331 (4.2)	10202 (4.3)	4872 (4.3)	1479 (4.2)	765 (5.0)
Unknown	126 (0.6)	1021 (0.7)	2226 (0.8)	2323 (1.0)	1327 (1.2)	360 (1.0)	121 (0.8)
Annual household income, yuan							
<10000	4844 (22.3)	33008 (23.3)	62013 (23.1)	51605 (21.9)	23731 (21.1)	6953 (19.9)	2993 (19.7)
10000-50000	11864 (54.7)	77442 (54.7)	146397 (54.6)	128621 (54.6)	60436 (53.9)	18752 (53.7)	8146 (53.7)
>50000	2988 (13.8)	19403 (13.7)	36710 (13.7)	34349 (14.6)	17311 (15.4)	5754 (16.5)	2603 (17.2)
Unknown	1982 (9.1)	11766 (8.3)	23087 (8.6)	21168 (9.0)	10743 (9.6)	3484 (10.0)	1414 (9.3)

© 2024 Chen L et al. *JAMA Network Open.*

Marital status							
Married	19575 (90.3)	128371 (90.6)	242844 (90.5)	212256 (90.0)	100620 (89.7)	31251 (89.4)	13699 (90.4)
Unmarried	1946 (9.0)	12143 (8.6)	23046 (8.6)	21066 (8.9)	10226 (9.1)	3312 (9.5)	1351 (8.9)
Unknown	157 (0.7)	1105 (0.8)	2317 (0.9)	2421 (1.0)	1375 (1.2)	380 (1.1)	106 (0.7)
Health insurance status							
Insured	21335 (98.4)	139083 (98.2)	262982 (98.1)	230618 (97.8)	109553 (97.6)	34216 (97.9)	14857 (98.0)
Uninsured	34 (0.2)	259 (0.2)	576 (0.2)	565 (0.2)	289 (0.3)	99 (0.3)	38 (0.3)
Unknown	309 (1.4)	2277 (1.6)	4649 (1.7)	4560 (1.9)	2379 (2.1)	628 (1.8)	261 (1.7)
Life behavior							
Current smoker	6309 (29.1)	44525 (31.4)	80169 (29.9)	64473 (27.3)	28031 (25.0)	8069 (23.1)	4030 (26.6)
Current drinker	6106 (28.2)	40868 (28.9)	77722 (29.0)	64702 (27.4)	28728 (25.6)	8349 (23.9)	4152 (27.4)
Lipids							
LDL, mg/dL	33.4 (27, 37.2)	58.8 (51.3, 64.8)	85.9 (78.5, 93)	113.2 (106.4, 120.9)	141.3 (135.1, 149)	170.4 (164.6, 178.2)	209.1 (197.1, 233.1)
HDL, mg/dL	51.8 (42.2, 65.7)	46 (37.9, 57.2)	48 (40.2, 58.4)	49.5 (42.2, 59.2)	51 (43.3, 60.7)	52.6 (44.1, 62.6)	53.8 (42.9, 67.3)
TC, mg/dL	110.6 (100.2, 123.4)	131.5 (118.7, 145)	162 (149.7, 174.8)	192.6 (180.2, 205.7)	223.5 (211.1, 237.8)	256 (242.8, 271.1)	303.9 (281.9, 341.1)
TG, mg/dL	145.3 (92.1, 246.2)	119.6 (84.1, 186)	130.2 (93, 191.3)	139.1 (100.1, 195.7)	146.1 (108.1, 201.1)	153.2 (112.5, 209.9)	159.4 (110.7, 238.3)
SBP, mmHg	152.5 (139.5, 166)	151 (139, 164.5)	151.5 (139, 165)	151.5 (139, 165)	152 (139.5, 165.5)	153.5 (140.5, 167)	153 (140, 167)
DBP, mmHg	86 (78, 95)	86 (78.5, 94.5)	86 (78.5, 94)	86 (79, 94)	86 (79, 94)	86.5 (79, 94)	87 (79, 95)
BMI, kg/m^2	25.4 (23, 27.9)	25.4 (23.1, 27.8)	25.6 (23.3, 27.9)	25.8 (23.6, 28.1)	25.8 (23.7, 28.2)	25.9 (23.7, 28.2)	25.7 (23.5, 27.9)
Waist, cm	88 (81, 95)	88 (81, 95)	88 (82, 95)	88 (82, 95)	89 (83, 95)	89 (83, 95)	88 (82, 95)
Medical History							

© 2024 Chen L et al. *JAMA Network Open.*

Diabetes	5176 (23.9)	28160 (19.9)	49059 (18.3)	43278 (18.4)	21777 (19.4)	7301 (20.9)	3213 (21.2)
Obesity	5304 (24.5)	33547 (23.7)	65838 (24.5)	61284 (26.0)	29921 (26.7)	9356 (26.8)	3734 (24.6)
Cancer	120 (0.6)	689 (0.5)	1231 (0.5)	1113 (0.5)	571 (0.5)	211 (0.6)	99 (0.7)
COPD	106 (0.5)	845 (0.6)	1088 (0.4)	847 (0.4)	342 (0.3)	113 (0.3)	48 (0.3)
CKD	47 (0.2)	336 (0.2)	620 (0.2)	540 (0.2)	318 (0.3)	157 (0.4)	81 (0.5)
Lipid-lowering treatment	2389 (11.0)	10899 (7.7)	12208 (4.6)	8495 (3.6)	4377 (3.9)	1741 (5.0)	1001 (6.6)

LDL=Low-density lipoprotein cholesterol. HDL= High-density lipoprotein. TC=Total cholesterol. TG=Triglyceride.

SBP=Systolic blood pressure. DBP= Diastolic blood pressure. BMI=Body mass index. COPD=chronic obstructive pulmonary disease.

© 2024 Chen L et al. *JAMA Network Open.*

eTable 3. Baseline characteristics of secondary prevention population.

	< 40 mg/dL (n=7,012)	40-70 mg/dL (n=31,572)	70-100 mg/dL (n=37,219)	100-130 mg/dL (n=27,332)	130-160 mg/dL (n=12,195)	160-190 mg/dL (n=4,064)	>190 mg/dL (n=1,710)
Age, y	62.2(8.1)	61.8(8.3)	61.8(8.2)	62(7.9)	62.3(7.7)	62.4(7.6)	62.3(7.7)
Sex							
Male	4470 (63.7)	19460 (61.6)	19221 (51.6)	11939 (43.7)	4615 (37.8)	1319 (32.5)	611 (35.7)
Female	2542 (36.3)	12112 (38.4)	17998 (48.4)	15393 (56.3)	7580 (62.2)	2745 (67.5)	1099 (64.3)
Urbanity							
Urban	3165 (45.1)	13391 (42.4)	14528 (39.0)	10637 (38.9)	4768 (39.1)	1760 (43.3)	692 (40.5)
Rural	3847 (54.9)	18181 (57.6)	22691 (61.0)	16695 (61.1)	7427 (60.9)	2304 (56.7)	1018 (59.5)
Region							
South	3090 (44.1)	14289 (45.3)	17364 (46.7)	13263 (48.5)	5883 (48.2)	2057 (50.6)	911 (53.3)
North	3922 (55.9)	17283 (54.7)	19855 (53.3)	14069 (51.5)	6312 (51.8)	2007 (49.4)	799 (46.7)
Education							
Primary school	2916 (41.6)	13782 (43.7)	18088 (48.6)	13700 (50.1)	6133 (50.3)	1968 (48.4)	901 (52.7)
Middle school	2372 (33.8)	10347 (32.8)	11212 (30.1)	7799 (28.5)	3481 (28.5)	1174 (28.9)	472 (27.6)
High school	1144 (16.3)	4983 (15.8)	5372 (14.4)	3984 (14.6)	1799 (14.8)	648 (15.9)	244 (14.3)
College or above	526 (7.5)	2213 (7.0)	2165 (5.8)	1593 (5.8)	664 (5.4)	243 (6.0)	80 (4.7)
Unknown	54 (0.8)	247 (0.8)	382 (1.0)	256 (0.9)	118 (1.0)	31 (0.8)	13 (0.8)
Annual household income, yuan							
<10000	1186 (16.9)	6081 (19.3)	8060 (21.7)	5915 (21.6)	2650 (21.7)	712 (17.5)	335 (19.6)
10000-50000	3909 (55.7)	17170 (54.4)	20414 (54.8)	15062 (55.1)	6588 (54.0)	2269 (55.8)	959 (56.1)
>50000	1385 (19.8)	5763 (18.3)	5956 (16.0)	4297 (15.7)	2049 (16.8)	767 (18.9)	295 (17.3)

© 2024 Chen L et al. *JAMA Network Open.*

Unknown	532 (7.6)	2558 (8.1)	2789 (7.5)	2058 (7.5)	908 (7.4)	316 (7.8)	121 (7.1)
Marital status							
Married	6452 (92.0)	29092 (92.1)	33929 (91.2)	24661 (90.2)	10930 (89.6)	3655 (89.9)	1548 (90.5)
Unmarried	508 (7.2)	2258 (7.2)	2957 (7.9)	2405 (8.8)	1146 (9.4)	378 (9.3)	153 (8.9)
Unknown	52 (0.7)	222 (0.7)	333 (0.9)	266 (1.0)	119 (1.0)	31 (0.8)	9 (0.5)
Health insurance status							
Insured	6927 (98.8)	31093 (98.5)	36618 (98.4)	26652 (97.5)	11785 (96.6)	4004 (98.5)	1689 (98.8)
Uninsured	11 (0.2)	46 (0.1)	49 (0.1)	42 (0.2)	23 (0.2)	8 (0.2)	4 (0.2)
Unknown	74 (1.1)	433 (1.4)	552 (1.5)	638 (2.3)	387 (3.2)	52 (1.3)	17 (1.0)
Life behavior							
Current smoker	1717 (24.5)	7716 (24.4)	8597 (23.1)	5697 (20.8)	2328 (19.1)	708 (17.4)	300 (17.5)
Current drinker	1847 (26.3)	7646 (24.2)	8926 (24.0)	6198 (22.7)	2520 (20.7)	833 (20.5)	360 (21.1)
Lipids							
LDL, mg/dL	33.8(28.4, 37.4)	56.3(48.6, 63.3)	84.6(77.4, 92.1)	112.8(106.1, 120.7)	141(134.8, 148.6)	170.6(165, 178.4)	208.6(197.2, 229.7)
HDL, mg/dL	51.4(43.3, 63)	46.4(38.7, 57.6)	49.1(41, 59.9)	51(42.5, 61.5)	51.8(43.3, 62.3)	53.8(45.6, 64.2)	55.3(44.5, 69.2)
TC, mg/dL	105.2(100.2, 117.6)	127.2(114.8, 141.5)	160.9(148.9, 174)	193(181, 206.1)	224.3(211.5, 238.6)	257(243.6, 271.5)	303.6(282.7, 337.6)
TG, mg/dL	117.8(80.6, 183.3)	110.7(81.5, 159.4)	125.8(91.2, 180.7)	136.4(99.2, 191.3)	147(108.1, 197.5)	150.6(113.4, 201.9)	159.4(115.1, 240)
SBP, mmHg	140(127.5, 154)	140(127.5, 155)	143(130, 159)	145(131, 161)	146.5(132.5, 162)	145(132, 160.5)	147(132.5, 162)
DBP, mmHg	82.3(12)	82.7(12)	83.9(12)	84.6(12.1)	84.7(11.9)	84.1(11.7)	84.6(12.2)
	81.5(74, 89.5)	82(74.5, 90)	83(75.5, 91.5)	84(76.5, 92)	84(76.5, 92.5)	83(76, 91.5)	84(76.5, 92)
BMI, kg/m^2	25.4(23.2, 27.7)	25.5(23.2, 27.7)	25.4(23.2, 27.7)	25.4(23.3, 27.8)	25.5(23.4, 27.9)	25.4(23.4, 27.6)	25.4(23.1, 27.9)

© 2024 Chen L et al. *JAMA Network Open.*

Waist, cm	88(81, 94)	88(81, 94.2)	87(80, 94)	87(80, 93)	87(80, 93.4)	87(80, 93)	87(80, 93.2)
Medical History							
Diabetes	1584 (22.6)	6330 (20.0)	6415 (17.2)	4564 (16.7)	2185 (17.9)	753 (18.5)	339 (19.8)
Obesity	1542 (22.0)	7199 (22.8)	8480 (22.8)	6321 (23.1)	2927 (24.0)	906 (22.3)	414 (24.2)
Cancer	33 (0.5)	198 (0.6)	260 (0.7)	173 (0.6)	64 (0.5)	37 (0.9)	8 (0.5)
COPD	69 (1.0)	234 (0.7)	276 (0.7)	176 (0.6)	70 (0.6)	19 (0.5)	7 (0.4)
CKD	55 (0.8)	250 (0.8)	314 (0.8)	282 (1.0)	130 (1.1)	62 (1.5)	26 (1.5)
Lipid-lowering treatment	2768 (39.5)	9748 (30.9)	6774 (18.2)	3370 (12.3)	1416 (11.6)	505 (12.4)	271 (15.8)

LDL=Low-density lipoprotein cholesterol. HDL= High-density lipoprotein. TC=Total cholesterol. TG=Triglyceride.

SBP=Systolic blood pressure. DBP= Diastolic blood pressure. BMI=Body mass index. COPD=chronic obstructive pulmonary disease.

© 2024 Chen L et al. *JAMA Network Open.*

eTable 4. Distribution of causes of death, overall and by different ASCVD risk groups.

Cause of death	Low-risk population (%)	Primary prevention (%)	Secondary prevention (%)	Total (%)
**All cause deaths**	44977	41217	6694	92888
**Level 2 Classification (ICD codes)**				
Cancers (C)	17005 (37.8)	12193 (29.6)	1459 (21.8)	30657 (33)
Blood and endocrine (D50-89, E)	1253 (2.8)	1892 (4.6)	276 (4.1)	3421 (3.7)
Mental and behavioural (F)	110 (0.2)	69 (0.2)	8 (0.1)	187 (0.2)
Neurological (G)	441 (1)	314 (0.8)	44 (0.7)	799 (0.9)
Cardiovascular disease (I)	15331 (34.1)	19341 (46.9)	3955 (59.1)	38627 (41.6)
Respiratory (J23-99)	2600 (5.8)	2338 (5.7)	278 (4.2)	5216 (5.6)
Liver cirrhosis (K70.3/71.7/74.3-6)	97 (0.2)	58 (0.1)	8 (0.1)	163 (0.2)
Digestive (K, ex cirrhosis)	1172 (2.6)	767 (1.9)	87 (1.3)	2026 (2.2)
Musculoskeletal (M)	176 (0.4)	90 (0.2)	19 (0.3)	285 (0.3)
Urologenital (N)	530 (1.2)	492 (1.2)	60 (0.9)	1082 (1.2)
Accidental – transport related (V)	1765 (3.9)	815 (2)	104 (1.6)	2684 (2.9)
Accidental ex transport (W/X00-59)	1762 (3.9)	884 (2.1)	135 (2)	2781 (3)
Self-harm/violence (X60-Y09)	710 (1.6)	345 (0.8)	57 (0.9)	1112 (1.2)
Other	2025 (4.5)	1619 (3.9)	204 (3)	3848 (4.1)
**Level 3 Classification (ICD codes)**				
Oesophageal cancer (C15)	856 (1.9)	730 (1.8)	79 (1.2)	1665 (1.8)
Stomach cancer (C16)	1730 (3.8)	1379 (3.3)	147 (2.2)	3256 (3.5)
Colorectal cancer (C18-21)	1232 (2.7)	971 (2.4)	104 (1.6)	2307 (2.5)
Liver cancer (C22)	2717 (6)	1626 (3.9)	172 (2.6)	4515 (4.9)
Pancriatic cancer (C25)	789 (1.8)	659 (1.6)	97 (1.4)	1545 (1.7)
Lung cancer (C34)	4935 (11)	3928 (9.5)	478 (7.1)	9341 (10.1)
Malignant melanoma (C43)	33 (0.1)	18 (0)	1 (0)	52 (0.1)
Female breast cancer (C50)	562 (1.2)	167 (0.4)	29 (0.4)	758 (0.8)
Uterus (C54-55)	173 (0.4)	73 (0.2)	9 (0.1)	255 (0.3)
Ovarian cancer (C56)	221 (0.5)	86 (0.2)	9 (0.1)	316 (0.3)
Prostate cancer (C61)	101 (0.2)	152 (0.4)	15 (0.2)	268 (0.3)
Kidney cancer (C64)	88 (0.2)	93 (0.2)	11 (0.2)	192 (0.2)
Bladder cancer (C67)	107 (0.2)	157 (0.4)	17 (0.3)	281 (0.3)
Brain/CNS cancer (C71-72)	437 (1)	241 (0.6)	43 (0.6)	721 (0.8)
Haematological malignancy (C81-96)	881 (2)	554 (1.3)	73 (1.1)	1508 (1.6)
Diabetes mellitus (E10-14)	1030 (2.3)	1696 (4.1)	242 (3.6)	2968 (3.2)
Dementia/Alzheimer (F00/01/03, G30)	79 (0.2)	94 (0.2)	10 (0.1)	183 (0.2)
Hypertensive heart disease (I11)	1009 (2.2)	1419 (3.4)	232 (3.5)	2660 (2.9)
Ischaemic heart disease (I20-25)	5584 (12.4)	7234 (17.6)	1632 (24.4)	14450 (15.6)
Atrial fibrillation/flutter (I48)	14 (0)	8 (0)	4 (0.1)	26 (0)
Heart failure (I50)	68 (0.2)	104 (0.3)	11 (0.2)	183 (0.2)
Stroke (I60-I64)	6862 (15.3)	8821 (21.4)	1759 (26.3)	17442 (18.8)
Aortic dissection (I71.0)	60 (0.1)	70 (0.2)	10 (0.1)	140 (0.2)

© 2024 Chen L et al. *JAMA Network Open.*

Aortic aneurysm (I71.1-9)	62 (0.1)	52 (0.1)	6 (0.1)	120 (0.1)
Peripheral vascular disease (I73)	6 (0)	3 (0)	0 (0)	9 (0)
Lower respiratory infection (J09-22)	466 (1)	502 (1.2)	57 (0.9)	1025 (1.1)
Falls (W00-19)	811 (1.8)	508 (1.2)	75 (1.1)	1394 (1.5)
Suicide (X60-84)	688 (1.5)	337 (0.8)	57 (0.9)	1082 (1.2)
Other	13376 (29.7)	9535 (23.1)	1315 (19.6)	24226 (26.1)

ICD-10=International Classification Diseases, 10th revision.

© 2024 Chen L et al. *JAMA Network Open.*

eTable 5. Associations of LDL-C levels with all cause, CVD, and cancer mortality in different ASCVD risk groups (mg/dL)

	< 40 mg/dL	40-70 mg/dL	70-100 mg/dL	100-130 mg/dL	130-160 mg/dL	160-190 mg/dL	>190 mg/dL
**No. of patients**							
Low-risk population	116991	592787	977570	730335	300866	84369	35436
Primary prevention	21678	141619	268207	235743	112221	34943	15156
Secondary prevention	7012	31572	37219	27332	12195	4064	1710
**All cause death**							
No. of event (Event rate per 1000 person-years)							
Low-risk population	2503 (5.4)	11230 (4.3)	15140 (3.4)	10203 (3.1)	4187 (3.2)	1127 (3.1)	587 (3.9)
Primary prevention	1228 (15.4)	8015 (13.7)	13562 (11.6)	11070 (10.8)	4991 (10.4)	1563 (10.7)	788 (13.2)
Secondary prevention	320 (12)	1672 (12.6)	2084 (12.5)	1543 (12.6)	729 (13.6)	224 (12.7)	122 (16.8)
**CVD death**							
No. of event (Event rate per 1000 person-years)							
Low-risk population	898 (2)	3798 (1.5)	5052 (1.1)	3562 (1.1)	1407 (1.1)	387 (1.1)	227 (1.5)
Primary prevention	580 (7.3)	3658 (6.2)	6277 (5.4)	5207 (5.1)	2440 (5.1)	774 (5.3)	405 (6.8)
Secondary prevention	172 (6.5)	933 (7)	1226 (7.4)	961 (7.8)	451 (8.4)	139 (7.9)	73 (10.1)
**Cancer death**							

© 2024 Chen L et al. *JAMA Network Open.*

No. of event (Event rate per 1000 person-years)							
Low-risk population	857 (1.9)	4052 (1.6)	5784 (1.3)	3980 (1.2)	1674 (1.3)	460 (1.3)	198 (1.3)
Primary prevention	354 (4.4)	2343 (4)	4136 (3.5)	3309 (3.2)	1444 (3)	412 (2.8)	195 (3.3)
Secondary prevention	85 (3.2)	425 (3.2)	443 (2.7)	314 (2.6)	132 (2.5)	40 (2.3)	20 (2.8)

© 2024 Chen L et al. *JAMA Network Open.*

eTable 6. Estimated change points in the association between LDL-C and mortality in different ASCVD risk groups, and associations with mortality below and above the change point, from piecewise two-line models.

	LDL-C(mg/dL) change point (95%CI)	HR(95%CI) per 1mmol/L increase below change point	HR(95%CI) per 1mmol/L increase above change point
**Overall**			
**Low-risk population**			
All cause mortality	105.60(103.42-107.77)	0.78(0.77-0.80)	1.07(1.05-1.10)
CVD mortality	110.33(107.21-113.43)	0.75(0.73-0.78)	1.11(1.06-1.16)
**Primary prevention**			
All cause mortality	122.88(119.22-126.55)	0.86(0.84-0.87)	1.11(1.08-1.14)
CVD mortality	136.50(131.74-141.26)	0.89(0.87-0.91)	1.18(1.12-1.23)
**Secondary prevention**			
All cause mortality	106.73(103.51-109.96)	1.02(0.96-1.08)	1.10(1.04-1.16)
CVD mortality	54.49(52.30-56.68)	0.77(0.53-1.12)	1.14(1.09-1.18)
**DM**			
**Low-risk population**			
All cause mortality	141.83(138.90-144.76)	0.89(0.84-0.94)	1.29(1.11-1.49)
CVD mortality	133.63(130.07-137.20)	0.99(0.88-1.10)	1.36(1.10-1.68)
**Primary prevention**			
All cause mortality	115.55(112.26-118.84)	0.94(0.90-0.99)	1.15(1.09-1.21)
CVD mortality	111.92(105.81-118.02)	1.01(0.94-1.09)	1.17(1.09-1.25)
**Secondary prevention**			
All cause mortality	51.23(49.72-52.73)	0.78(0.41-1.49)	1.26(1.20-1.33)
CVD mortality	42.85(41.31-44.40)	0.29(0.08-0.99)	1.29(1.21-1.37)
**Non-DM**			
**Low-risk population**			
All cause mortality	100.29(98.41-102.11)	0.77(0.75-0.79)	1.06(1.03-1.09)
CVD mortality	124.16(120.41-128.05)	0.78(0.76-0.81)	1.22(1.14-1.3)
**Primary prevention**			
All cause mortality	114.14(110.41-117.68)	0.81(0.79-0.83)	1.1(1.06-1.14)
CVD mortality	134.04(129.41-138.54)	0.85(0.82-0.88)	1.2(1.13-1.28)
**Secondary prevention**			
All cause mortality	120.30(115.41-125.57)	0.96(0.9-1.02)	1.05(0.94-1.16)
CVD mortality	31.65(18.41-44.714)	0.59(0.07-5.25)	1.08(1.03-1.13)

HR=hazard ratio. CI=confidence interval. CVD=cardiovascular disease. The multivariable adjusted analyses utilized the variables in Model3.

© 2024 Chen L et al. *JAMA Network Open.*

eFigure 1. The flowchart of study design.

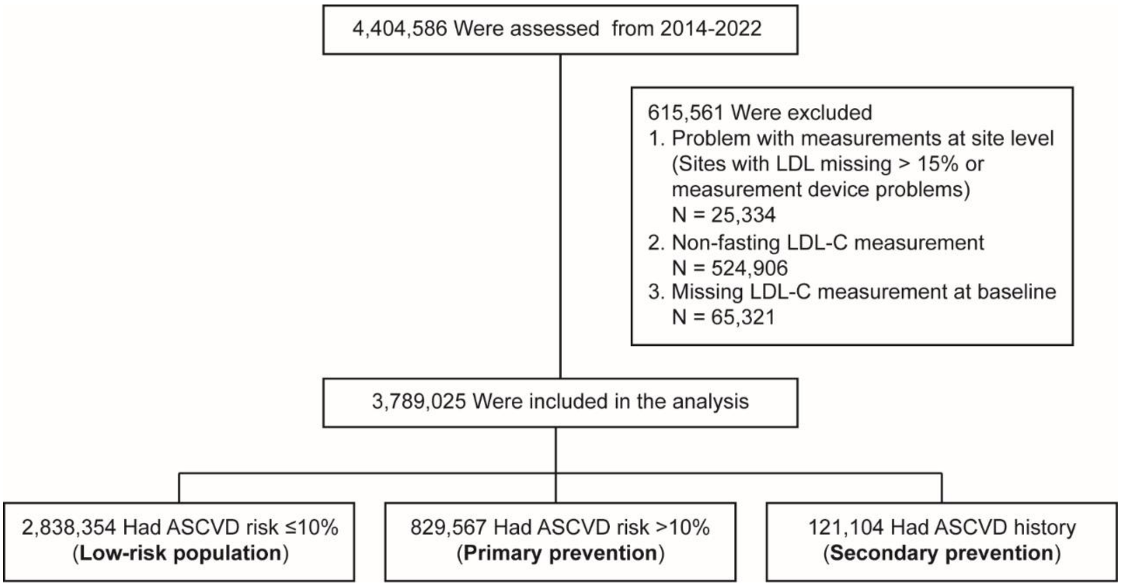

© 2024 Chen L et al. *JAMA Network Open.*

eFigure 2. Baseline LDL-C distribution in different subgroups.

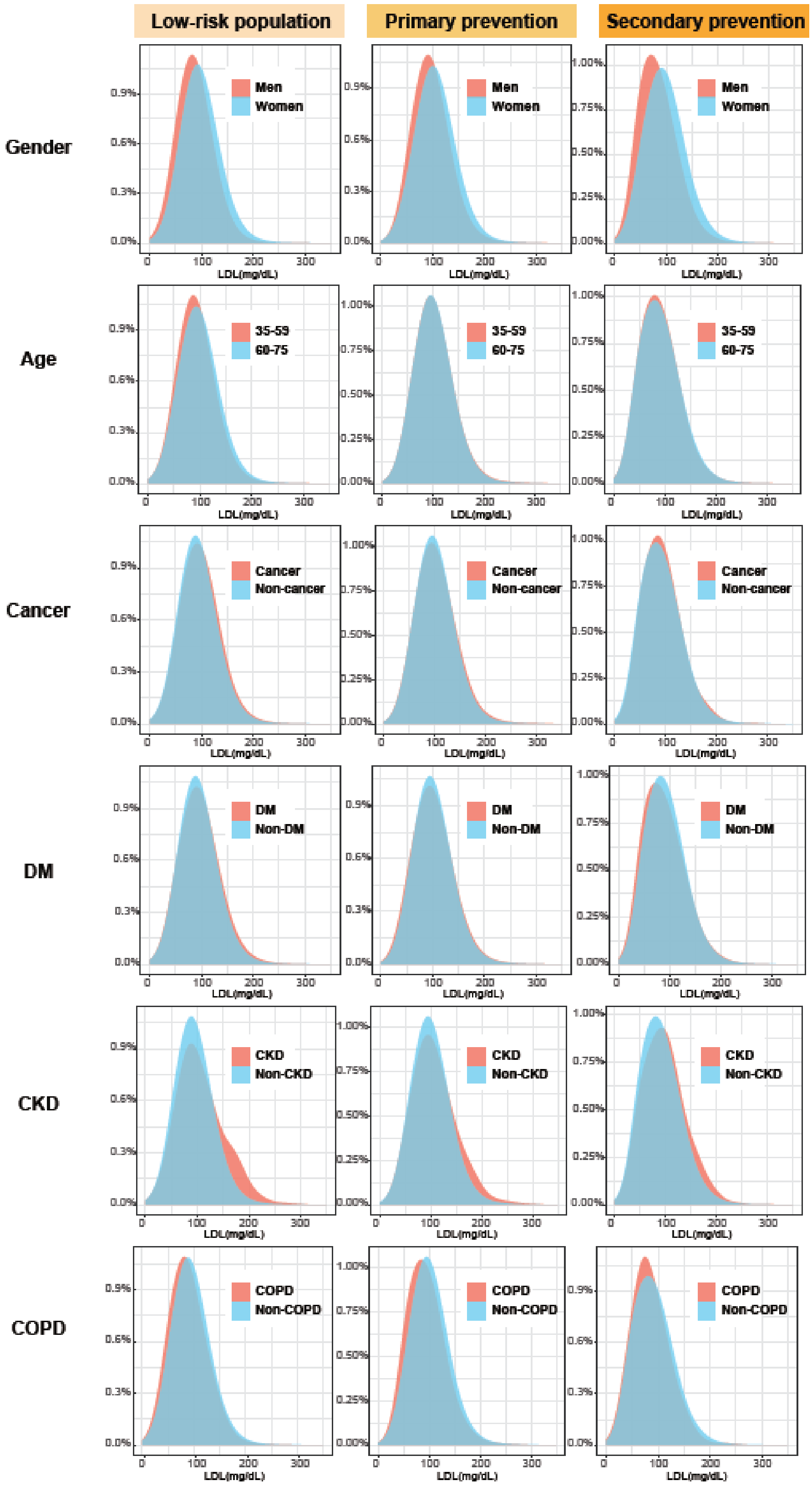

DM=diabetes. CKD= chronic kidney disease. COPD= chronic obstructive pulmonary disease.

© 2024 Chen L et al. *JAMA Network Open.*

eFigure 3. Hazard ratio for all cause (A) and CVD (B) mortality according to categories of LDL-C with different covariates adjusted.

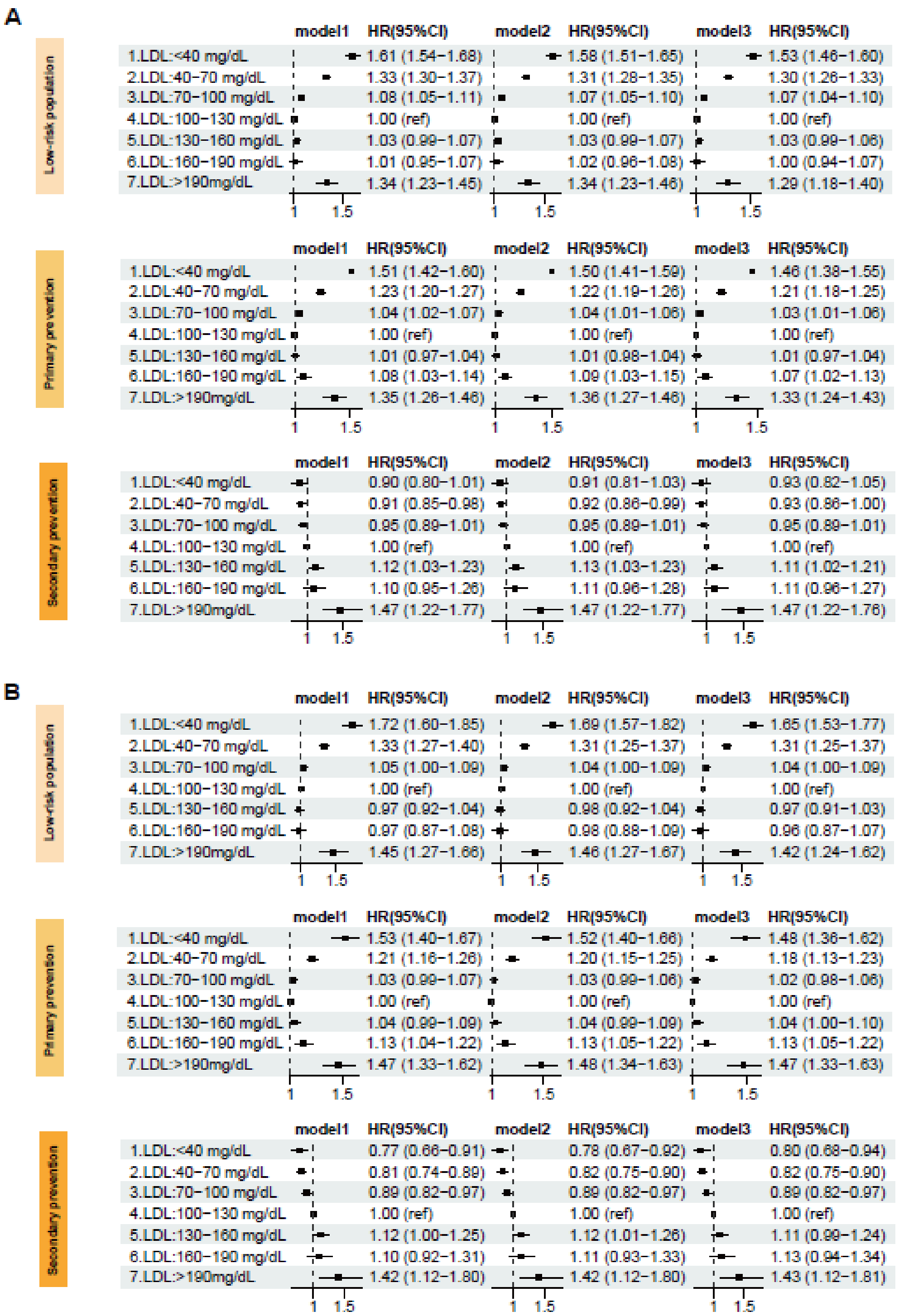

HR=hazard ratio. CI=confidence interval. CVD=cardiovascular disease.

Model1: age + sex

Model2: age + sex + education + annual household income

Model3: age + sex + education + annual household income + chronic obstructive pulmonary disease + cancer + diabetes + current smoking + alcohol status + obesity + systolic blood pressure + lipid-lowering treatment + high-density lipoprotein + triglyceride.

© 2024 Chen L et al. *JAMA Network Open.*

eFigure 4. Association between LDL-C and all cause (A), CVD (B) mortality in different ASCVD risk groups by sex.

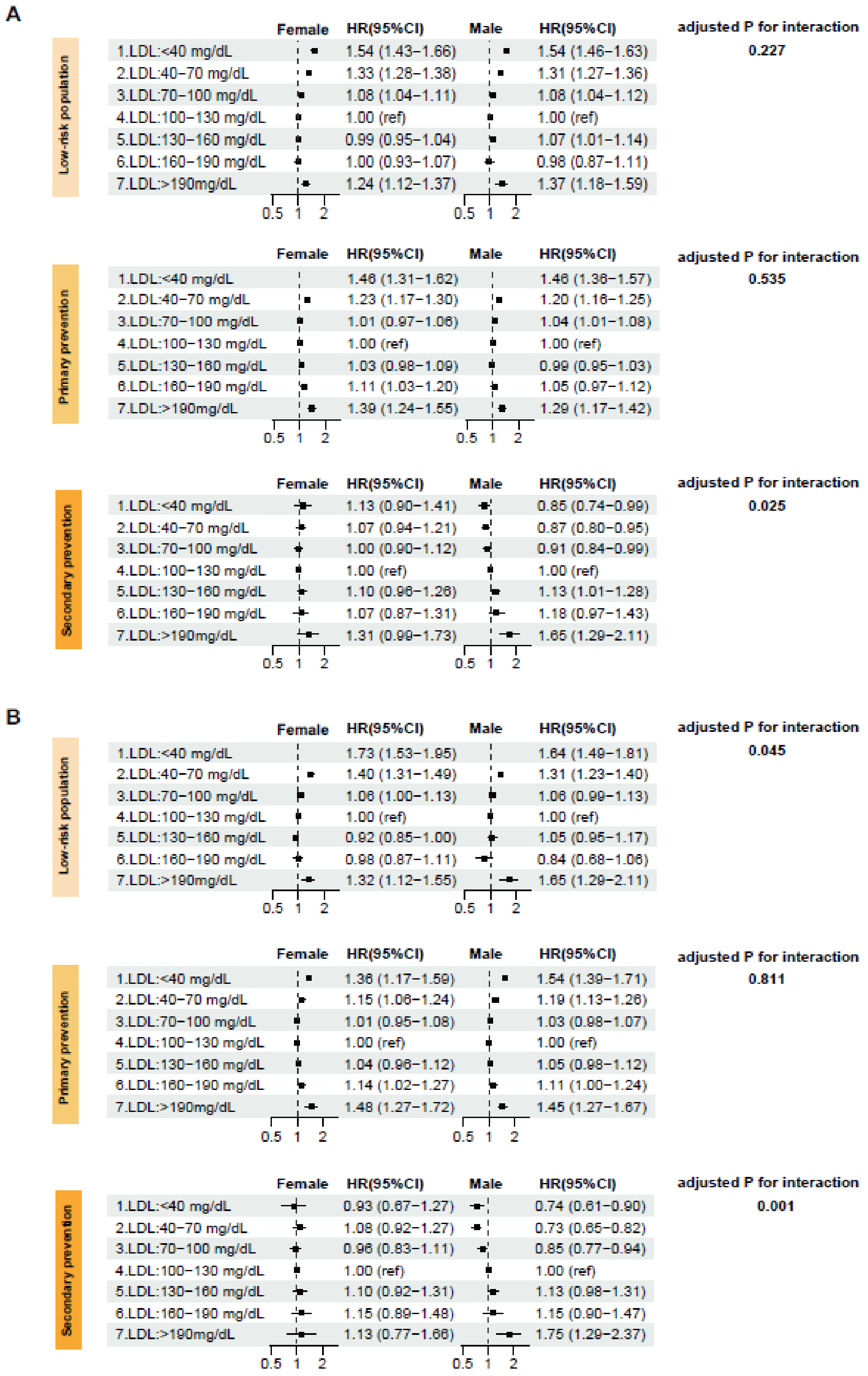

HR=hazard ratio. CI=confidence interval. CVD=cardiovascular disease. The multivariable adjusted analyses utilized the variables in Model3 except sex.

© 2024 Chen L et al. *JAMA Network Open.*

eFigure 5. Association between LDL-C and all cause (A), CVD (B) mortality in different ASCVD risk groups by age.

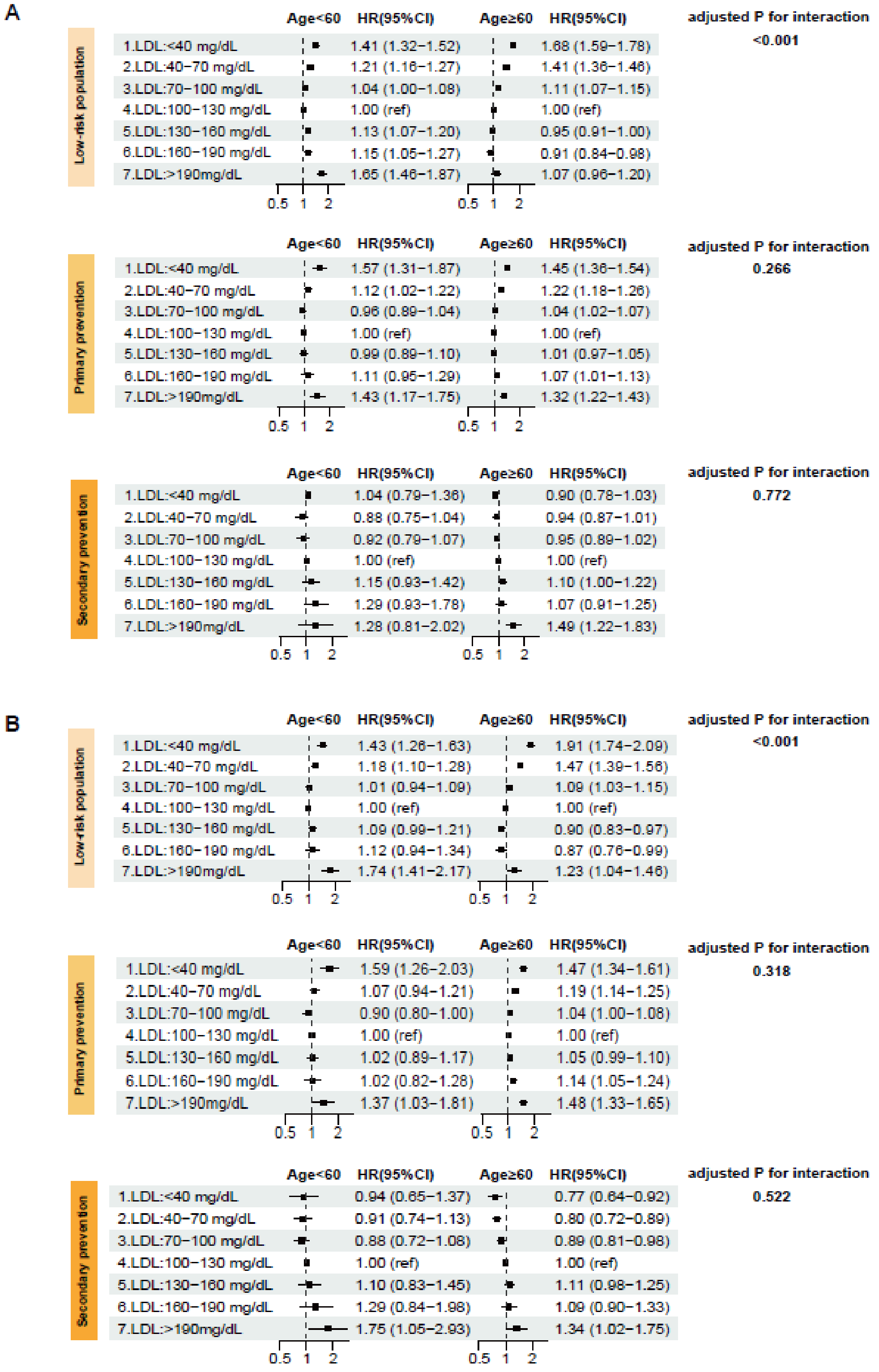

HR=hazard ratio. CI=confidence interval. CVD=cardiovascular disease. The multivariable adjusted analyses utilized the variables in Model3 except age.

© 2024 Chen L et al. *JAMA Network Open.*

eFigure 6. Association between LDL-C and all cause (A), CVD (B) mortality in different ASCVD risk groups by hypertension.

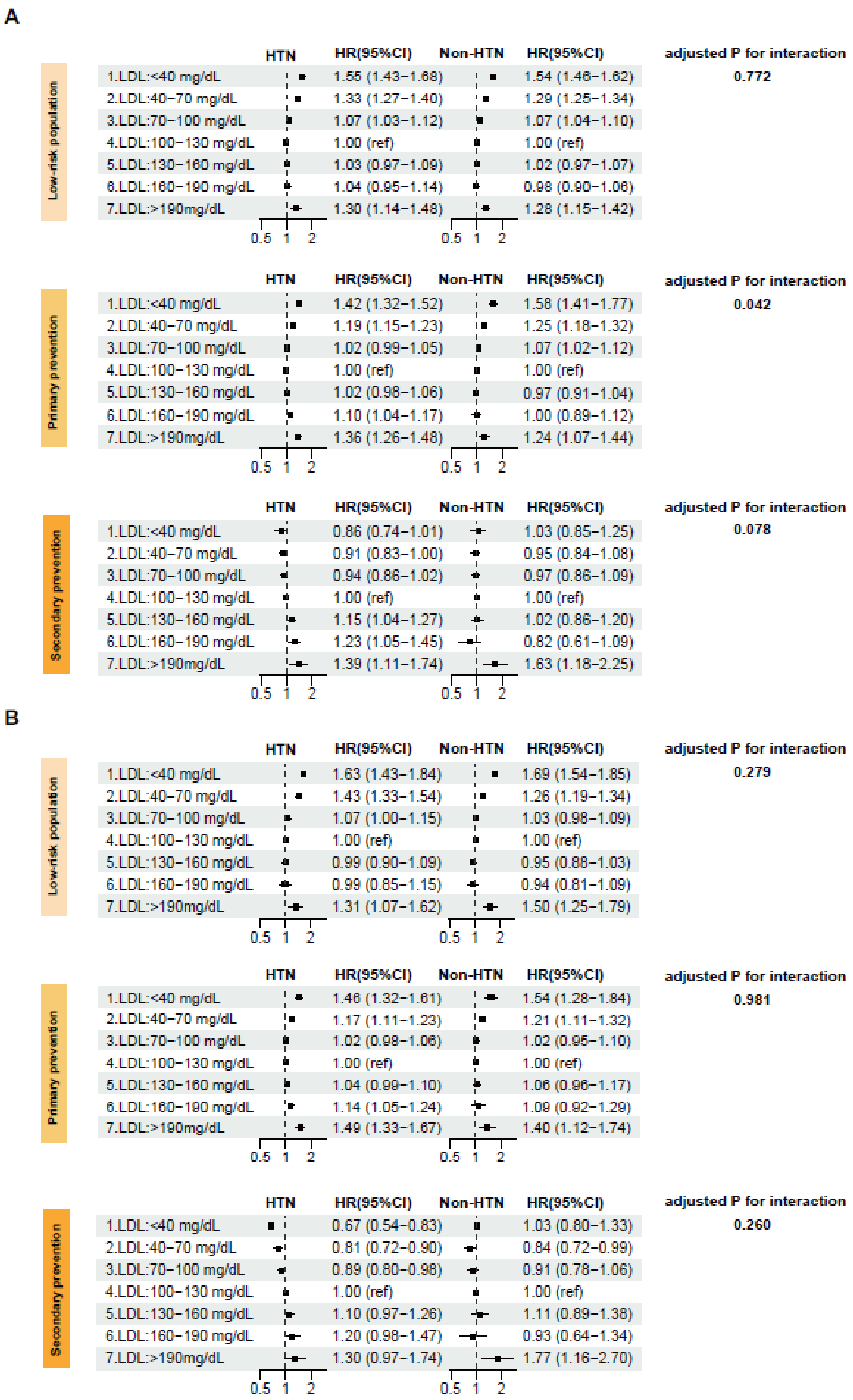

HR=hazard ratio. CI=confidence interval. CVD=cardiovascular disease. HTN=hypertension. The multivariable adjusted analyses utilized the variables in Model3 except systolic blood pressure

© 2024 Chen L et al. *JAMA Network Open.*

eFigure 7. Associations between LDL-C and all-cause and CVD mortality stratified by diabetes status.

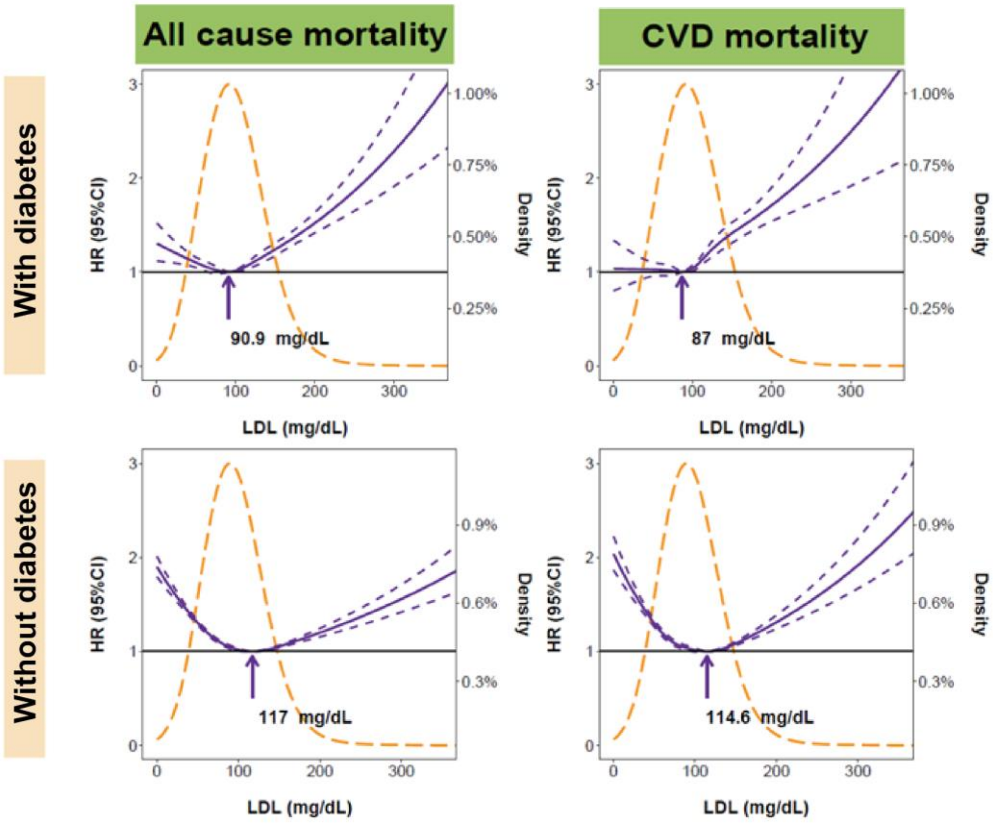

HR=hazard ratio. CI=confidence interval. CVD=cardiovascular disease. Multivariable adjusted hazard ratios for all cause, CVD and cancer mortality according to levels of LDL-C on a continuous scale. Solid purple lines are multivariable adjusted hazard ratios, with dashed lines showing 95% confidence interval derived from restricted cubic spline regressions with four knots. Dashed yellow curves show fraction of population with different level of LDL-C. Arrows indicate the concentration of LDL-C with the lowest risk of mortality. Analyses utilized the variables in Model3.

© 2024 Chen L et al. *JAMA Network Open.*

eFigure 8. Spline models of the association between LDL-C and all-cause, CVD, and cancer mortality in different ASCVD risk groups.

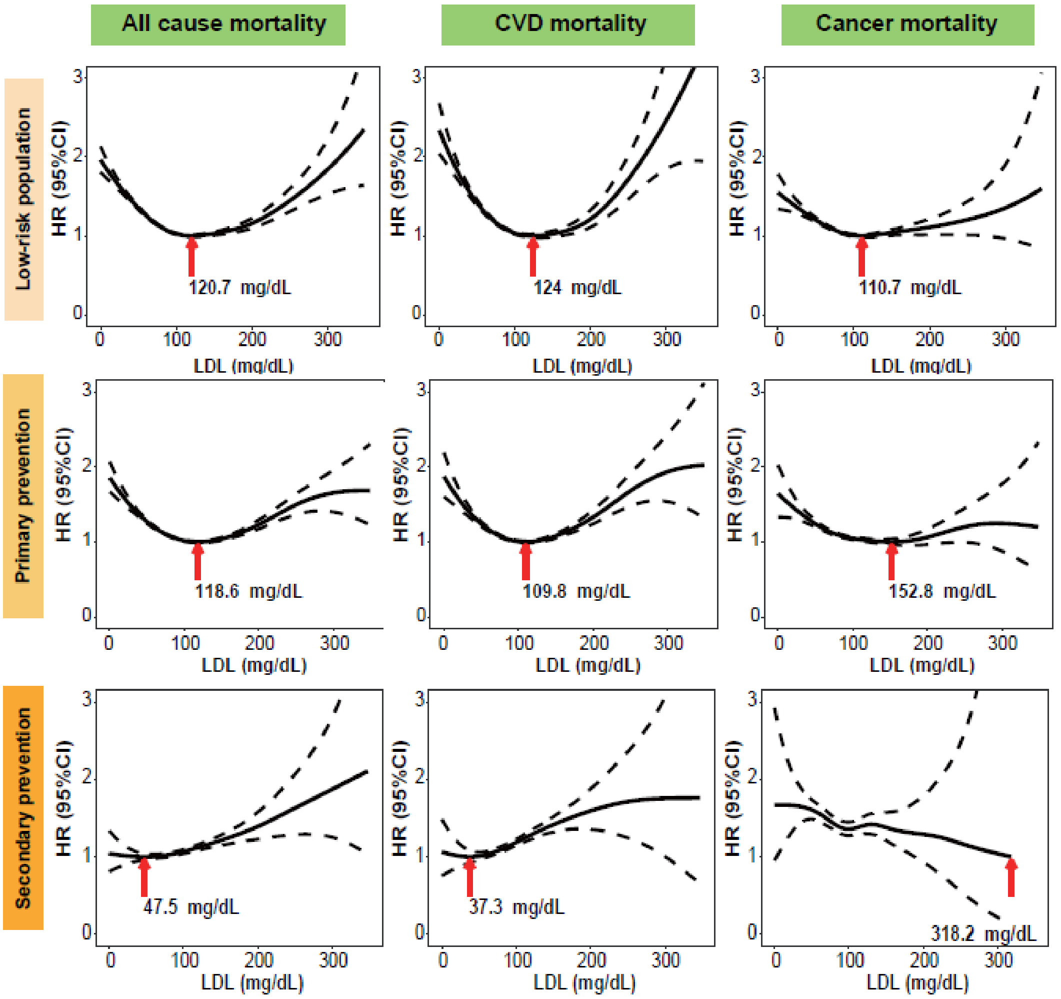

HR=hazard ratio. CI=confidence interval. CVD=cardiovascular disease. Analyses utilized the variables in Model3.

© 2024 Chen L et al. *JAMA Network Open.*

eFigure 9. Association between LDL-C and all cause mortality in different ASCVD risk groups exclusion of individual with baseline chronical disease.

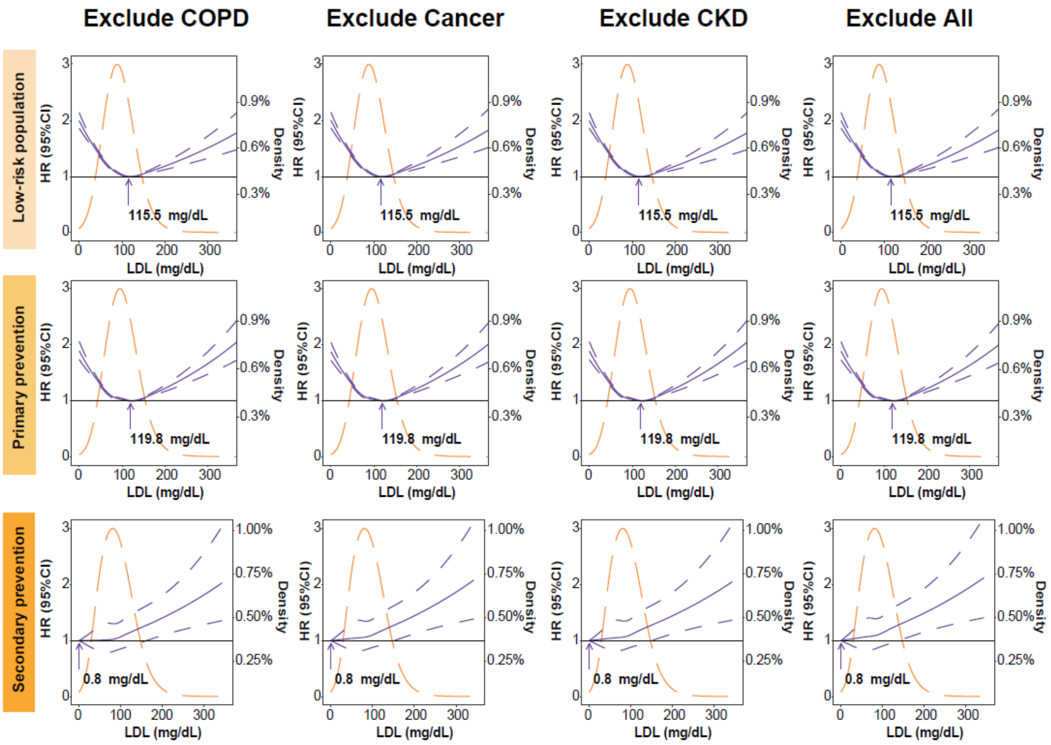

HR=hazard ratio. CI=confidence interval. COPD= chronic obstructive pulmonary disease. CKD= chronic kidney disease. Analyses utilized the variables in Model3.

© 2024 Chen L et al. *JAMA Network Open.*

eFigure 10. Association between LDL-C and all cause, CVD, and cancer mortality in different ASCVD risk groups after exclusion of individual with less than 3-year follow-up.

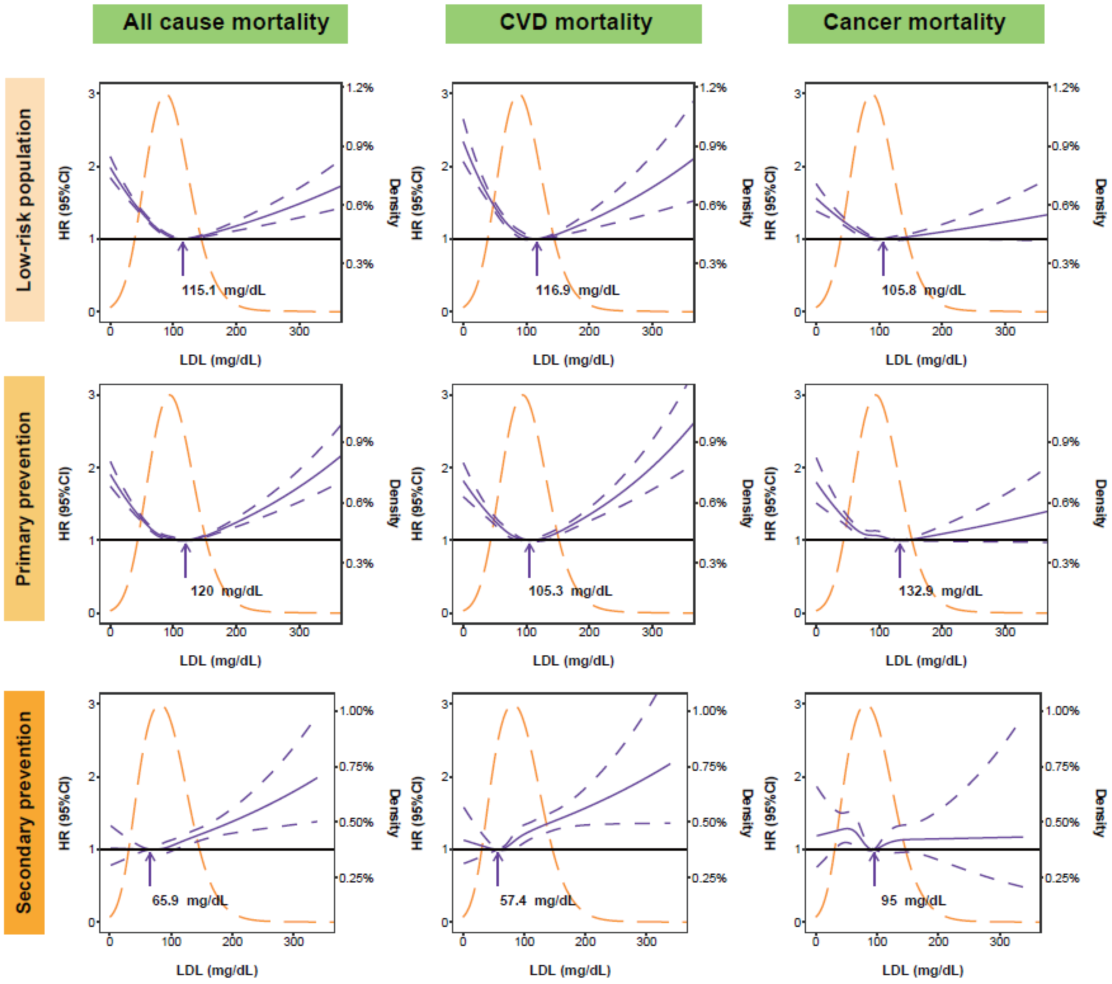

HR=hazard ratio. CI=confidence interval. CVD=cardiovascular disease. Multivariable adjusted hazard ratios for all cause, CVD and cancer mortality according to levels of LDL-C on a continuous scale. Solid purple lines are multivariable adjusted hazard ratios, with dashed lines showing 95% confidence interval derived from restricted cubic spline regressions with four knots. Dashed yellow curves show fraction of population with different level of LDL-C. Arrows indicate the concentration of LDL-C with the lowest risk of mortality. Analyses utilized the variables in Model3.

© 2024 Chen L et al. *JAMA Network Open.*

eFigure 11. Association between adjusted LDL-C and all-cause, CVD, and cancer mortality in different ASCVD risk groups.

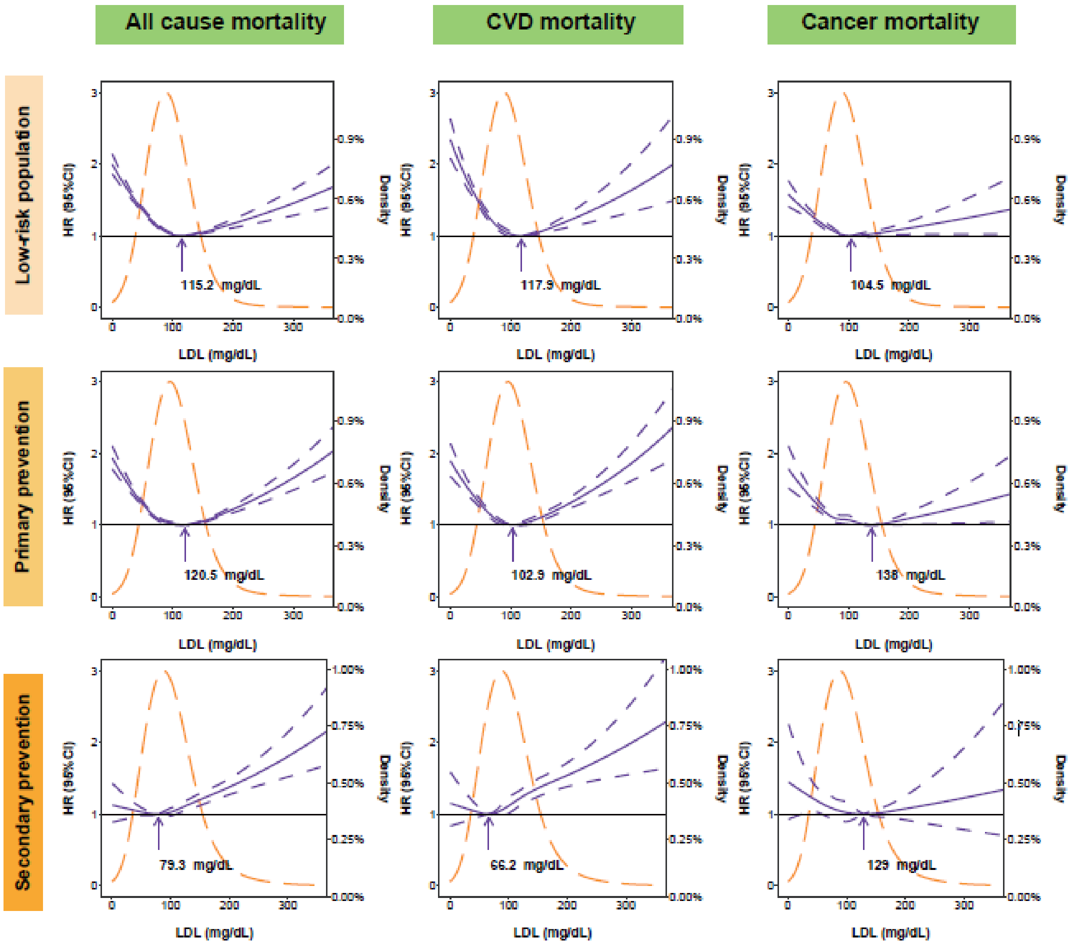

HR=hazard ratio. CI=confidence interval. CVD=cardiovascular disease. Multivariable adjusted hazard ratios for all cause, CVD and cancer mortality according to levels of LDL-C on a continuous scale. Solid purple lines are multivariable adjusted hazard ratios, with dashed lines showing 95% confidence interval derived from restricted cubic spline regressions with four knots. Dashed yellow curves show fraction of population with different level of LDL-C. Arrows indicate the concentration of LDL-C with the lowest risk of mortality. Analyses utilized the variables in Model3. LDL-C was multiplied by 1.43 in those using cholesterol lowering drugs, and multiplied by 1.67 in those using cholesterol lowering drugs with concurrent diagnoses of ischemic heart disease or stroke.

© 2024 Chen L et al. *JAMA Network Open.*

eFigure 12. Association between LDL-C and all-cause, CVD, and cancer mortality in different ASCVD risk groups after excluding lipid-lowering treatment.

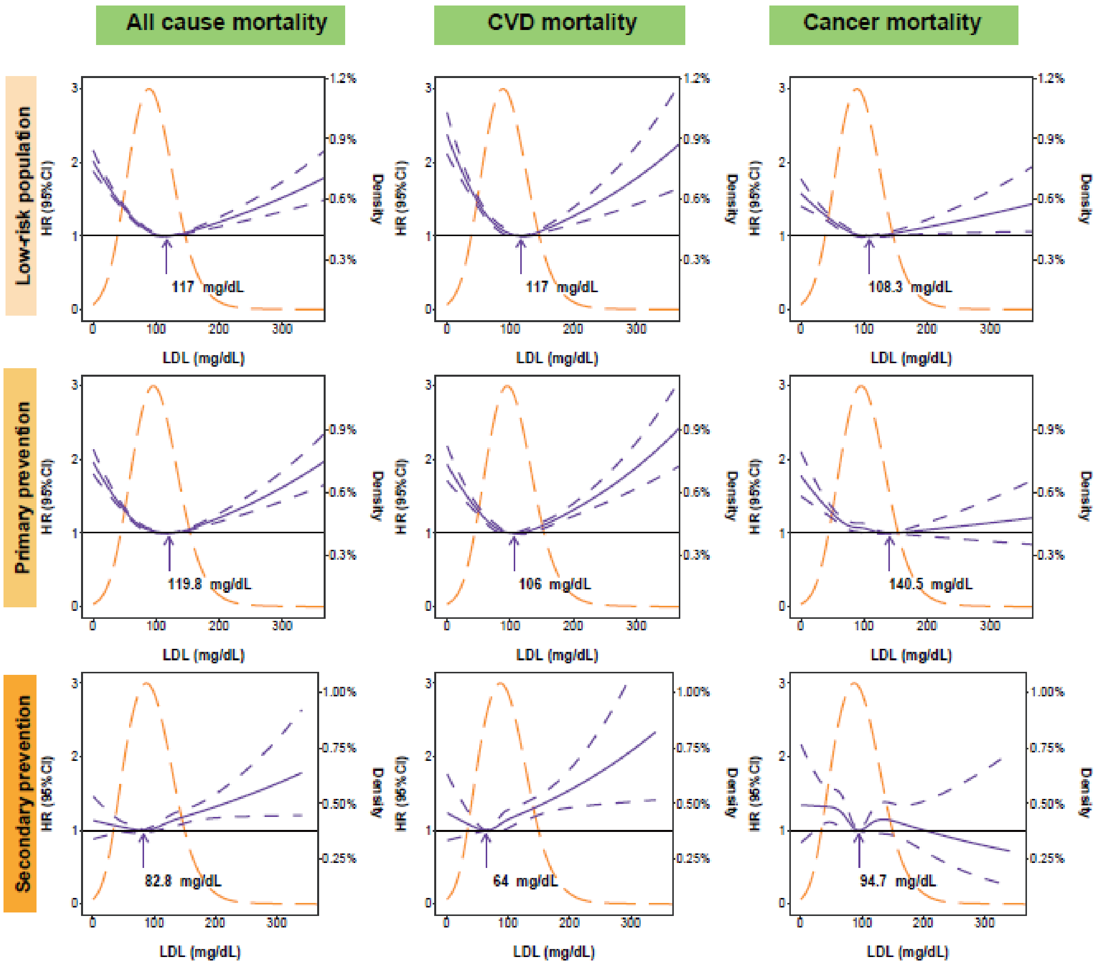

HR=hazard ratio. CI=confidence interval. CVD=cardiovascular disease. Multivariable adjusted hazard ratios for all cause, CVD and cancer mortality according to levels of LDL-C on a continuous scale. Solid purple lines are multivariable adjusted hazard ratios, with dashed lines showing 95% confidence interval derived from restricted cubic spline regressions with four knots. Dashed yellow curves show fraction of population with different level of LDL-C. Arrows indicate the concentration of LDL-C with the lowest risk of mortality. Analyses utilized the variables in Model3 except lipid-lowering treatment.

eReferences

1. Sampson M, Ling C, Sun Q, Harb R, Ashmaig M, Warnick R, et al. A New Equation for Calculation of Low-Density Lipoprotein Cholesterol in Patients With Normolipidemia and/or Hypertriglyceridemia. JAMA Cardiol. 2020;5(5):540-8.
2. Sakuma M, Iimuro S, Shinozaki T, Kimura T, Nakagawa Y, Ozaki Y, et al. Optimal target of LDL cholesterol level for statin treatment: challenges to monotonic relationship with cardiovascular events. BMC Medicine. 2022;20(1):441.

© 2024 Chen L et al. *JAMA Network Open.*
