# Supplementary material for: Low-Density Lipoprotein Cholesterol, Cardiovascular Disease Risk, and Mortality in China
Source: JAMA Netw Open. 2024 Jul 18;7(7):e2422558. doi: 10.1001/jamanetworkopen.2024.22558 (PMC11258592; doi:10.1001/jamanetworkopen.2024.22558)
Supplement: Supplement 2. — Data Sharing Statement [file jamanetwopen-e2422558-s002.pdf]

Data Sharing Statement

Chen. Low-Density Lipoprotein Cholesterol, Cardiovascular Disease Risk, and Mortality in China. *JAMA Netw Open*. Published July 18, 2024. doi:10.1001/jamanetworkopen.2024.22558

Data
Data available: No
